# Adipocyte G_i_ signaling is essential for maintaining whole-body glucose homeostasis and insulin sensitivity

**DOI:** 10.1038/s41467-020-16756-x

**Published:** 2020-06-12

**Authors:** Lei Wang, Sai P. Pydi, Lu Zhu, Luiz F. Barella, Yinghong Cui, Oksana Gavrilova, Kendra K. Bence, Cecile Vernochet, Jürgen Wess

**Affiliations:** 1 Molecular Signaling Section, Laboratory of Bioorganic Chemistry, National Institute of Diabetes and Digestive and Kidney Diseases, Bethesda, MD 20892 USA; 2 Mouse Metabolism Core, National Institute of Diabetes and Digestive and Kidney Diseases, Bethesda, MD 20892 USA; 3 Internal Medicine Research Unit, Worldwide Research, Development and Medical, Pfizer Inc, Cambridge, MA 02139 USA

**Keywords:** Metabolism, Medical research

## Abstract

Adipocyte dysfunction links obesity to insulin resistance and type 2 diabetes. Adipocyte function is regulated by receptor-mediated activation of heterotrimeric G proteins. Little is known about the potential in vivo metabolic roles of G_i_-type G proteins expressed by adipocytes, primarily due to the lack of suitable animal models. To address this question, we generated mice lacking functional G_i_ proteins selectively in adipocytes. Here we report that these mutant mice displayed significantly impaired glucose tolerance and reduced insulin sensitivity when maintained on an obesogenic diet. In contrast, using a chemogenetic strategy, we demonstrated that activation of G_i_ signaling selectively in adipocytes greatly improved glucose homeostasis and insulin signaling. We also elucidated the cellular mechanisms underlying the observed metabolic phenotypes. Our data support the concept that adipocyte G_i_ signaling is essential for maintaining euglycemia. Drug-mediated activation of adipocyte G_i_ signaling may prove beneficial for restoring proper glucose homeostasis in type 2 diabetes.

## Abstract (web summary)

Gs-coupled receptor signaling is well known to modulate adipocyte metabolism, but the role of Gi-coupled receptors in adipose tissue is less well understood. Here the authors show that signaling via Gi-type G proteins expressed by adipocytes is essential for maintaining proper blood glucose homeostasis.

## Introduction

Adipocytes play a central role in the pathogenesis of T2D^1^. In obese individuals, adipocytes undergo hypertrophy and macrophage infiltration, resulting in the secretion of inflammatory adipokines and the release of excessive amounts of free fatty acids (FFAs) into the circulation. These obesity-related metabolic disturbances eventually lead to peripheral insulin resistance and impaired glucose homeostasis^2–5^.

The function of adipocytes, like that of virtually all other cell types, is regulated by the activity of cell surface receptors that belong to the superfamily of G protein-coupled receptors (GPCRs)^6,7^. These receptors are coupled to one or more of the three major G protein families, G_s_, G_i_, and G_q_^6,7^.

The role of G_s_-coupled receptors in modulating adipocyte function and whole-body glucose homeostasis has been studied in considerable detail^8–13^. In contrast to adipocyte G_s_, little is known about the physiological and pathophysiological roles of G_i_-type G proteins expressed by adipose tissue. Like virtually all other GPCRs, the G_i_-linked GPCRs expressed by adipocytes, including the CB_1_ cannabinoid and HCA_2_ (GPR109A) receptors^7,14–16^, are also expressed in many other tissues and cell types. Thus, it is not possible to assess the in vivo metabolic roles of G_i_-coupled receptors expressed by adipocytes by simply monitoring the in vivo effects of receptor subtype-selective agonists or antagonists.

It should also be noted that the G_i_ family of heterotrimeric G proteins (Gα subunits) consists of several molecularly distinct members which are widely expressed throughout the body (α_i1_, α_i2_, α_i3_, and α_o_)^17^. Additional members of this protein family show a more restricted expression pattern, including α_t_ (transducin), α_gust_ (gustducin), and α_z_^17^. Because of this multiplicity of G_i_-type G proteins, it is extremely challenging to study the physiological roles of G_i_ signaling by using gene knockout technology.

To circumvent these difficulties, we expressed the catalytic subunit of pertussis toxin (S1-PTX) selectively in mouse adipocytes, resulting in mutant mice lacking functional G_i_ proteins only in adipocytes (“adipo-Gi KO mice”; note that PTX selectively inactivates all Gα_i/o_ subunits, except α_z_, by covalent modification of a C-terminal Cys residue^17^).

We also generated a complimentary mouse model expressing a G_i_-coupled designer GPCR (G_i_ DREADD; G_i_-Coupled Designer Receptor Exclusively Activated by a Designer Drug; better known as hM4D or GiD)^18,19^ selectively in adipocytes. Treatment of these mutant mice (adipo-GiD mice) with clozapine-*N*-oxide (CNO), a compound that is otherwise pharmacologically inert^18,19^, triggers the selective activation of adipocyte G_i_ signaling.

Surprisingly, adipo-Gi KO mice show significant impairments in glucose homeostasis on an obesogenic diet, while G_i_ activation in adipocytes of adipo-GiD mice leads to improved glucose tolerance and insulin sensitivity. These data indicate that adipocyte G_i_ signaling is essential for maintaining proper blood glucose homeostasis and that drugs able to selectively stimulate G_i_ signaling in adipocytes may prove useful for the treatment of T2D and related metabolic disorders.

## Results

### Mice lacking functional Gα_i_ proteins in adipocytes

Mouse adipose tissues express multiple G_i_ proteins (Gα-subunits; G_αi1_, G_αi2_, Gα_i3_, and Gα_o_) (Supplementary Fig. 1). To inactivate the function of these G proteins, we expressed the S1 subunit of PTX selectively in adipocytes by introducing the *adipoq-Cre* transgene^20^ into the genome of *ROSA26*^*PTX*^ mice^21^ (Supplementary Fig. 2a, b). Throughout the text, we refer to these mice simply as ‘adipo-Gi KO mice’. *ROSA26*^*PTX*^ littermates that did not harbor the *adipoq-Cre* transgene served as control animals throughout all experiments. Unless stated otherwise, all studies were carried out with adult male mice that were at least 8 weeks old (genetic background: C57BL/6).

Both in vivo (Supplementary Fig. 2c) and in vitro (Supplementary Fig. 3a, b) functional studies confirmed that the expression of PTX in adipocytes of adipo-Gi KO mice inactivated G_i_-type G proteins. The mRNA levels of all major G protein α- and βγ-subunits and selected adipocyte G_i_- and G_s_-coupled receptors were not significantly different between control and adipo-Gi KO adipocytes (Supplementary Fig. 4a–e). However, expression of the metabolically important β3-aderengic receptor, a G_s_-coupled receptor, trended to be higher in the KO adipocytes (*P* = 0.06; *n* = 4 per group, two-tailed Mann–Whitney test) (Supplementary Fig. 4e).

### Lack of adipocyte G_i_ signaling increases lipolysis

**Fig. 1:**
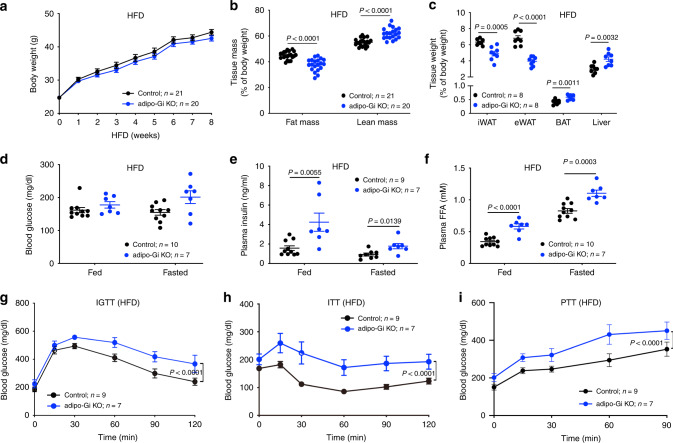
Mice lacking G_i_ signaling in adipocytes show metabolic impairments when maintained on a HFD. **a** Body weight growth curves of control and adipo-Gi KO mice (males) maintained on a HFD. **b** Fat and lean body mass of control and adipo-Gi KO mice maintained on HFD for 8 weeks. **c** Weight of inguinal white adipose tissue (iWAT), epididymal white adipose tissue (eWAT), brown adipose tissue (BAT), and liver of control and adipo-Gi KO mice fed with HFD for 8 weeks. **d**–**f** Freely fed and fasting blood glucose (**d**), plasma insulin (**e**), and plasma free fatty acid (FFA) levels (**f**) of control and adipo-Gi KO mice maintained on HFD for 8 weeks. **g**–**i** In vivo metabolic tests carried out with control and adipo-Gi KO mice maintained on HFD for 8 weeks. **g** I.p. glucose tolerance test (1 g/kg glucose; IGTT). **h** Insulin tolerance test (1 U/kg insulin, i.p.; ITT). **i** Pyruvate tolerance test (2 g/kg pyruvate, i.p.; PTT). Data are given as means ± s.e.m. *P* values are indicated in the different panels (**a**, **g**–**i**: two-way ANOVA followed by Bonferroni’s post hoc test; **b**–**f**: two-tailed Student’s *t* test). Source data are provided as a Source data file.

To study the potential in vivo roles of adipocyte G_i_ signaling, we maintained adipo-Gi KO and control mice on either regular chow (RC) or a high-fat diet (HFD, 60% calories from fat). Adipo-Gi KO and control mice did not exhibit any significant differences in body weight, independent of the diet that they consumed (Fig. 1a and Supplementary Fig. 2d). However, body fat mass was markedly decreased in the adipo-Gi KO mice, while lean body mass was significantly increased in these mutant mice (Fig. 1b and Supplementary Fig. 2e). In HFD adipo-Gi KO mice, the weight of inguinal white adipose tissue (iWAT) and epididymal WAT (eWAT) was markedly decreased (Fig. 1c). In contrast, the weight of brown adipose tissue (BAT) and liver weight were significantly increased in the HFD KO mice (Fig. 1c). The lack of adipocyte G_i_ signaling had no significant effect on fed and fasting blood glucose levels (Fig. 1d and Supplementary Fig. 2e). Interestingly, plasma insulin levels were increased in HFD adipo-Gi KO mice under both fed and fasting conditions (Fig. 1e), indicative of peripheral insulin resistance.

We also found that plasma FFA levels were significantly increased in both RC and HFD adipo-Gi KO mice, consistent with enhanced lipolysis (Fig. 1f and Supplementary Fig. 2e). These results suggest that lack of adipocyte G_i_ signaling promotes lipolysis, resulting in a decrease in body fat mass.

**Fig. 2:**
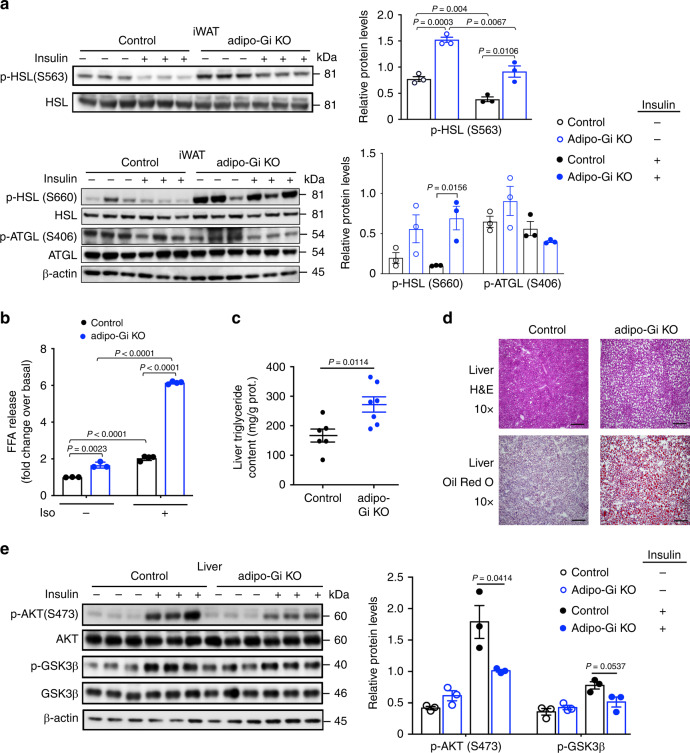
Lack of G_i_ signaling in adipocytes increases lipolysis and causes liver steatosis. **a** Western blotting analysis of p-HSL/HSL protein expression levels in iWAT prepared from HFD control and adipo-Gi KO mice. Mice (males) were injected with 5 U of insulin (i.v.), and iWAT was collected 5 min later (*n* = 3 per group). **b** In vitro lipolysis assay. Primary adipocytes prepared from iWAT of control and adipo-Gi KO mice were left either untreated (*n* = 3 per group) or incubated with 1 μM isoproterenol (Iso; *n* = 4 per group). **c**, Liver triglyceride content of HFD control and adipo-Gi KO mice (control, *n* = 6; adipo-Gi KO, *n* = 7). **d** Representative images of H&E- and Oil Red O-stained liver tissues of HFD control and adipo-Gi KO mice (*n* = 4 per group; scale bars = 200 μm). **e** Western blotting analysis of hepatic insulin signaling. HFD control and adipo-Gi KO mice were injected i.v. with either saline or insulin (5 U). Liver tissue was collected 5 min later and processed for immunoblotting studies (*n* = 3 per group). Data are given as means ± s.e.m. *P* values are indicated in the different panels. (**a**, **b**, **e**: two-way ANOVA followed by Bonferroni’s post hoc test; **c**: two-tailed Student’s *t* test). Source data are provided as a Source data file.

To confirm that the elevated plasma FFA levels caused by adipocyte G_i_ deficiency were due to increased adipose tissue lipolysis, we injected HFD adipo-Gi KO mice and control littermates with insulin (5 U/mouse i.v.) and collected iWAT tissue 5 min later. We then studied the expression levels of the phosphorylated (activated) form of hormone-sensitive lipase (p-HSL(S563) and p-HSL(S660)) via western blotting. Phosphorylation of HSL at S563 and S660 are critical for HSL activation and the breakdown of triglycerides^22^. We found that the expression levels of p-HSL(S563) and p-HSL(S660) were significantly elevated in iWAT from adipo-Gi KO mice, as compared with iWAT from control mice. This effect was observed under both basal conditions (after saline injection) and after insulin treatment (Fig. 2a). On the other hand, phosphorylation of adipose tissue triglyceride lipase (ATGL) at S406 was not enhanced in adipo-Gi KO mice (Fig. 2a). This observation was not unexpected since several studies suggest that PKA does not play a role in ATGL phosphorylation/activation^23^.

In parallel, we also performed in vitro lipolysis assays using primary adipocytes prepared from iWAT of adipo-Gi KO mice and control littermates. Even under basal conditions (no drug treatment), lipolysis (measured as release of FFA into the medium) was significantly increased in the mutant adipocytes (Fig. 2b). Treatment with isoproterenol (1 μM), a β-adrenergic receptor agonist, stimulated lipolysis in both mutant and control adipocytes (Fig. 2b). However, the amount of isoproterenol-induced FFA release was ~3-fold higher in the KO adipocytes, as compared to the corresponding control cells (Fig. 2b). Taken together, these data indicate that deficient adipocyte G_i_ function strongly promotes lipolysis in adipose tissue.

### Deficient adipocyte G_i_ function causes hepatic steatosis

We next examined whether increased plasma FFA levels caused ectopic lipid accumulation in the liver. Consistent with the observation that HFD adipo-Gi KO mice displayed an increase in liver weight (Fig. 1c), the mutant mice also showed a marked increase in liver triglyceride content (hepatic steatosis), as compared to HFD control littermates (Fig. 2c). This observation was confirmed by H&E and Oil Red O staining studies (Fig. 2d). To study hepatic insulin signaling at the molecular level, we injected HFD adipo-Gi KO mice and control littermates with saline or insulin (5 U/mouse i.v.) and collected liver tissue 5 min later. Immunoblotting studies indicated that the ability of insulin to phosphorylate AKT and GSK-3β was significantly reduced in the KO livers, indicative of hepatic insulin resistance (Fig. 2e). In sum, these data clearly indicate that the lack of adipocyte G_i_ signaling caused liver steatosis and hepatic insulin resistance.

### HFD adipo-Gi KO mice show impaired glucose homeostasis

To explore whether deficient adipocyte G_i_ signaling affected whole-body glucose homeostasis, we subjected both RC and HFD adipo-Gi KO mice and their control littermates to i.p. glucose, insulin, and pyruvate tolerance tests (IGTT, ITT, and PTT, respectively). RC mutant and control mice displayed similar glucose tolerance and insulin sensitivity (IGTT and ITT, respectively) (Supplementary Fig. 2f, g). In contrast, HFD adipo-Gi KO mice showed significantly impaired glucose tolerance and insulin sensitivity, as compared to their HFD control littermates (Fig. 1g, h). Moreover, the HFD KO mice displayed markedly enhanced blood glucose excursions in the PTT, consistent with increased hepatic glucose production (Fig. 1i). Taken together, these data indicate that lack of adipocyte G_i_ signaling caused impaired glucose homeostasis and insulin sensitivity when mice were metabolically challenged by an obesogenic, calorie-rich diet.

### Hyperinsulinemic-euglycemic clamp study

**Fig. 3:**
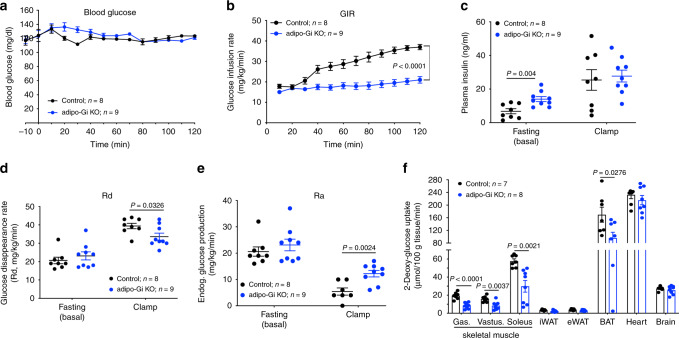
HFD adipo-Gi KO mice show severe insulin resistance in a hyperinsulinemic-euglycemic clamp study. Clamp studies were carried out with adipo-Gi KO mice and control littermates (males) that had been maintained on a HFD for 12 weeks. **a** Blood glucose levels during the course of the clamp study. **b** Glucose infusion rate (GIR). **c** Plasma insulin levels (basal state and during clamp). **d** Glucose disappearance rate (Rd). **e** Rate of endogenous glucose production rate (Ra). **f** Glucose uptake by gastrocnemius muscle (Gas.), vastus muscle (Vas.), soleus muscle, iWAT, eWAT, BAT, heart, and brain during the clamp. Data are given as means ± s.e.m. (*n* = 8 or 9 per group). *P* values are indicated in the different panels (**b**: two-way ANOVA followed by Bonferroni’s post hoc test; **c**–**f**: two-tailed Student’s *t* test). Source data are provided as a Source data file.

To further assess insulin sensitivity and glucose fluxes in HFD adipo-Gi KO and control mice, we performed a hyperinsulinemic-euglycemic clamp study (Fig. 3). Glucose infusion rate (GIR, an indicator of whole-body insulin action) was significantly decreased in HFD adipo-Gi KO mice, as compared with control littermates (Fig. 3a, b), indicative of severely impaired insulin sensitivity in the mutant mice. At the beginning of the clamp, plasma insulin levels were markedly increased in the mutant mice (also see Fig. 1e), while plasma insulin levels did not differ significantly between the two groups during the clamp (Fig. 3c). During the clamp, the rate of glucose disappearance rate (Rd) was significantly decreased in HFD adipo-Gi KO mice (Fig. 3d). The rate of endogenous glucose production (Ra), a parameter which primarily reflects hepatic glucose production^24^, was greatly enhanced in the adipo-Gi KO mice, indicative of hepatic insulin resistance (Fig. 3e).

To further clarify which additional tissues contributed to the impaired insulin sensitivity observed with adipo-Gi KO mice, we measured tissue-specific 2-deoxy-glucose uptake during the clamp. Under these conditions, adipo-Gi KO mice showed a marked reduction in glucose uptake into all three skeletal muscles investigated (gastrocnemius, vastus, and soleus muscles; Fig. 3f). Similarly, glucose uptake into BAT was significantly reduced in the mutant mice (Fig. 3f). These data indicate that HFD adipo-Gi KO mice suffer from severe insulin resistance, as demonstrated by increased GIR, elevated hepatic glucose production, and decreased glucose uptake into skeletal muscle tissues and BAT.

### Treatment of HFD adipo-Gi KO mice with an HSL inhibitor

**Fig. 4:**
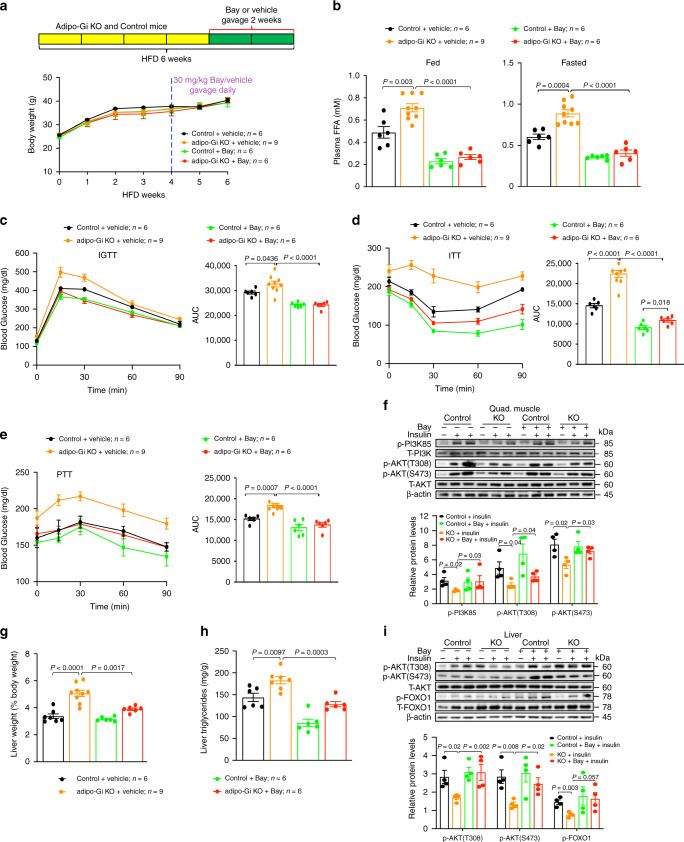
Inhibition of lipolysis prevents metabolic deficits caused by deficient adipocyte G_i_ signaling. **a** Scheme of the experimental design used (top). Control and adipo-Gi KO mice (males) consumed a HFD for 6 weeks and were treated once daily with Bay (30 mg/kg via oral gavage) or vehicle during the last 2 weeks of HFD feeding. Body weight growth curves are shown in the bottom panel. **b** Plasma free fatty acid (FFA) levels under fed and fasting conditions after vehicle or Bay treatment for 2 weeks (see the scheme in **a**). **c**–**e** In vivo metabolic tests after Bay treatment for 2 weeks. **c** Intraperitoneal glucose tolerance test (1 g/kg glucose, IGTT). **d** Insulin tolerance test (1 U/kg, i.p.; ITT). **e** Pyruvate tolerance test (2 g/kg pyruvate, i.p.; PTT). **f** Immunoblots showing insulin-induced phosphorylation of PI3K85, AKT-T308, and AKT- S473 (tissues were collected 5 min after i.v. injection of insulin or saline) (*n* = 4 per group). **g**, **h** Effect of Bay treatment (2 weeks) on liver weight (**g**) and hepatic triglyceride content (**h**). **i** Immunoblots showing insulin-induced phosphorylation of AKT-T308, AKT-S473, and FOXO1 in liver tissue after treatment of HFD mice with Bay or vehicle for 2 weeks. Liver tissue was collected 5 min after i.v. injection of insulin or saline (−) (*n* = 4 per group). *P* values are indicated in the different panels (two-way ANOVA followed by Bonferroni’s post hoc test test). AUC area under the curve. Source data are provided as a Source data file.

Elevated plasma FFA levels can cause insulin resistance and inflammation in the major insulin target tissues (including skeletal muscle and liver) and represent a key link between obesity, insulin resistance, inflammation, and the development of T2D^25^. To study whether the observed increases in plasma FFA levels were responsible for the multiple metabolic deficits displayed by the HFD adipo-Gi KO mice, we treated HFD adipo-Gi KO mice and control littermates with BAY 59–9435 (Bay), an HSL inhibitor that reduces lipolysis but does not affect body weight and food intake in mice^26,27^. Mice were maintained on the HFD for 6 weeks. During weeks 5 and 6, mice received either Bay (30 mg/kg) or vehicle via oral gavage (one dose per day; Fig. 4a). Chronic Bay treatment had no significant effect on body weight (Fig. 4a) or food intake (Supplementary Fig. 5). After Bay (or saline) treatment of mice for 2 weeks, plasma FFA levels remained markedly elevated in saline-treated adipo-Gi KO mice, whereas Bay-treated adipo-Gi KO mice showed greatly reduced plasma FFA levels, similar to those found with Bay-treated control mice (Fig. 4b). After chronic Bay (or saline) treatment for 2 weeks, HFD adipo-Gi KO mice and control littermates were subjected to a series of metabolic tests. In all tests (IGTT, ITT, and PTT), Bay-treated HFD adipo-Gi KO mice showed striking metabolic improvements, as compared to saline-treated HFD mutant mice (Fig. 4c–e). In fact, the glucose responses observed with this group of mice were not significantly differently from those obtained with control littermates (Fig. 4c–e).

In addition, chronic Bay treatment restored normal insulin signaling in skeletal muscle (quadriceps) prepared from HFD adipo-Gi KO mice (Fig. 4f) and markedly reduced liver weight and hepatic triglyceride content in this group of mice (Fig. 4g, h). Chronic Bay administration also completely restored control-like hepatic insulin signaling in HFD adipo-Gi KO mice (Fig. 4i).

In sum, these data strongly suggest that the elevated plasma FFA levels caused by the lack of adipocyte G_i_ signaling are the major contributor to the metabolic deficits displayed by the HFD adipo-Gi KO mice.

### Energy homeostasis is unchanged in HFD adipo-Gi KO mice

To study whether the lack of adipocyte G_i_ function affected energy expenditure, we employed indirect calorimetry to measure total energy expenditure (TEE) and related parameters in HFD adipo-Gi KO mice and control littermates. We found that both groups of mice showed similar TEE, respiratory exchange rate (RER), total locomotor activity, and cumulative food intake, either at room temperature (22 °C) or at thermoneutrality (30 °C) (Supplementary Fig. 6). Consistent with these data, the expression levels of genes coding for adipose tissue (iWAT, eWAT and BAT) beiging or browning and mitochondrial function were similar between HFD control and adipo-Gi KO mice (Supplementary Fig. 7a, b). Moreover, the protein expression levels of UCP-1 (BAT and iWAT) were not significantly altered by the lack of adipocyte G_i_ function (Supplementary Fig. 7c).

### Inflammatory cytokines are elevated in HFD adipo-Gi KO mice

**Fig. 5:**
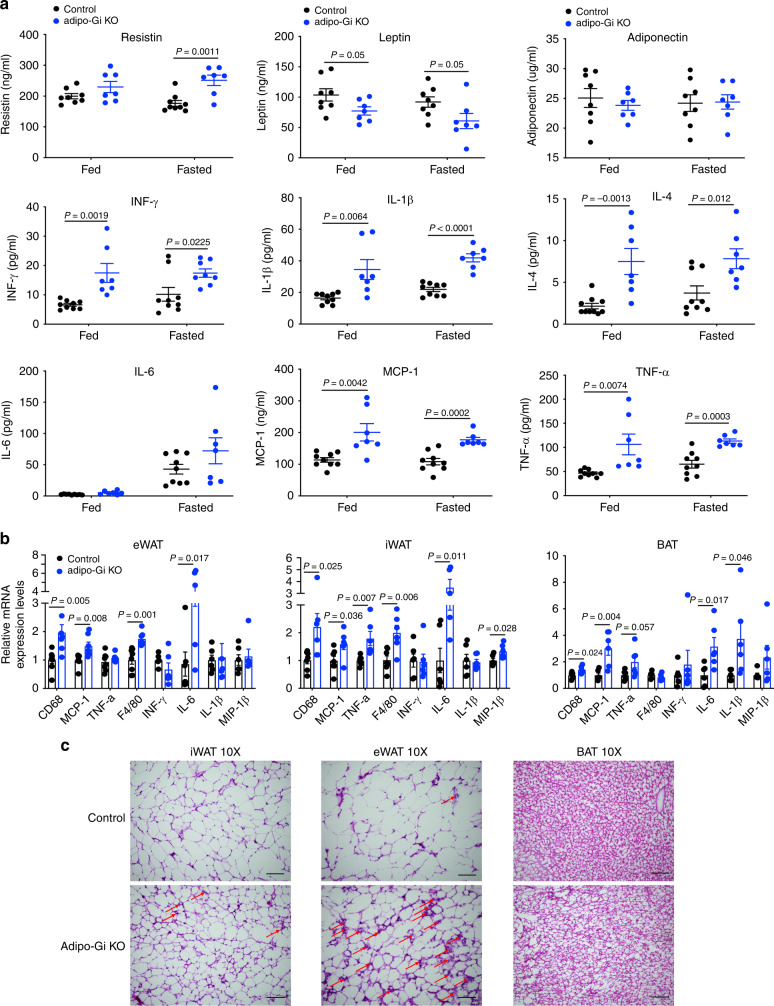
Plasma inflammatory cytokines and adipose tissue inflammation markers are increased in HFD adipo-Gi KO mice. **a** Plasma adipokine and cytokine levels of adipo-G_i_ KO and control mice (males) after 8 weeks of HFD feeding (control, *n* = 9; adipo-Gi KO, *n* = 7). Interferon-γ, INF-γ; interleukin-1 β, IL-1 β; interleukin-6, IL-6; interleukin-4, IL-4; monocyte chemoattractant protein-1, MCP-1; tumor necrosis factor α, TNF-α. **b** Relative mRNA expression levels of adipose tissue genes related to inflammation (*n* = 6 per group). **c** Representative Hematoxylin & Eosin (H&E) staining of sections of inguinal white adipose tissue (iWAT), epididymal WAT (eWAT), and brown adipose tissue (BAT) from mice maintained on a HFD for 12 weeks (*n* = 6 per group; scale bars = 200 μm). Representative mononuclear inflammatory cells are highlighted by red arrows. Data are given as means ± s.e.m. *P* values are indicated in the different panels (**a**, **b**: two-tailed Student’s *t* test). Source data are provided as a Source data file.

Adipocytes can secrete bioactive factors (adipokines/cytokines) that are able to modify the function of other metabolically important organs such as skeletal muscle, liver, heart, or endocrine pancreas^3,28–31^. Under pathophysiological conditions, pro-inflammatory adipokines (or cytokines) make a major contribution to peripheral insulin resistance and impaired glucose homeostasis^28–31^. To examine the potential involvement of adipocyte G_i_ signaling in this process, we measured plasma adipokine and cytokine levels in HFD adipo-Gi KO and control mice. Plasma leptin levels were significantly decreased in both RC and HFD adipo-Gi KO mice (Fig. 5a and Supplementary Fig. 2e), consistent with the observation that adipo-Gi KO mice showed decreased adiposity. Plasma adiponectin levels were similar in both groups of mice (Fig. 5a and Supplementary Fig. 2e). Interestingly, the plasma levels of resistin and several pro-inflammatory cytokines, including INF-γ, IL-1β, MCP-1, and TNF-α, were significantly elevated in HFD adipo-Gi KO mice under both fed and fasting conditions (Fig. 5a). Moreover, the mRNA expression levels of many inflammatory cytokines were significantly elevated in adipose tissues (iWAT, eWAT and BAT) from HFD adipo-Gi KO mice (Fig. 5b). H&E staining experiments indicated that adipocytes (iWAT and eWAT) from HFD adipo-Gi KO mice, despite their smaller size, were extensively infiltrated by mononuclear inflammatory cells (Fig. 5c). On the other hand, BAT cells from HFD adipo-Gi KO mice were visibly larger than control BAT adipocytes (Fig. 5c).

We also carried out immunostaining studies using an anti-F4/80 antibody to visualize macrophage infiltration of adipose tissue (iWAT and eWAT) in HFD adipo-Gi KO mice and control littermates. These studies demonstrated that macrophage infiltration was clearly increased in HFD adipo-Gi KO mice, as compared to HFD control littermates (Supplementary Fig. 8).

These data suggest that adipose tissue inflammation represents a key factor contributing to the metabolic deficits displayed by the HFD adipo-Gi KO mice.

### Bay Treatment of HFD adipo-Gi KO mice reduces inflammation

To examine the contribution of elevated plasma FFA levels to adipose tissue inflammation displayed caused by the lack of adipocyte Gi signaling, we measured plasma levels of inflammatory cytokines in adipo-Gi KO and control mice after 6 weeks of HFD feeding. During the last 2 weeks of HFD feeding, mice were treated daily with the HSL inhibitor Bay (30 mg/kg/day via oral gavage) or vehicle. We found that Bay treatment effectively reduced the elevated plasma cytokine levels (INF-γ, IL-10, IL-1β, IL-6, IL-4, and TNF-α) displayed by HFD adipo-Gi KO mice to levels similar to those observed with control mice (Supplementary Fig. 9a). Bay treatment of HFD adipo-Gi KO mice also reduced the elevated transcript levels of inflammatory cytokines in iWAT and eWAT to control levels (Supplementary Fig. 9b, c). H&E staining studies showed that BAY-treated HFD adipo-Gi KO mice restored control-like morphology to iWAT, eWAT, and BAT of HFD adipo-Gi KO mice (Supplementary Fig. 9d). Since Bay treatment of HFD adipo-Gi KO reduced plasma FFA levels to control-like levels (see Fig. 4b), it is likely that elevated FFA levels are the major trigger of the inflammatory processes observed with HFD adipo-Gi KO mice.

We also showed that Bay treatment dramatically reduced the number of F4/80-positive cells (macrophages) in adipose tissue (iWAT and eWAT) prepared from HFD adipo-Gi KO mice (Supplementary Fig. 8), linking elevated plasma FFA levels to increased macrophage infiltration.

### Reduced glucose uptake in HFD adipo-Gi KO mice

**Fig. 6:**
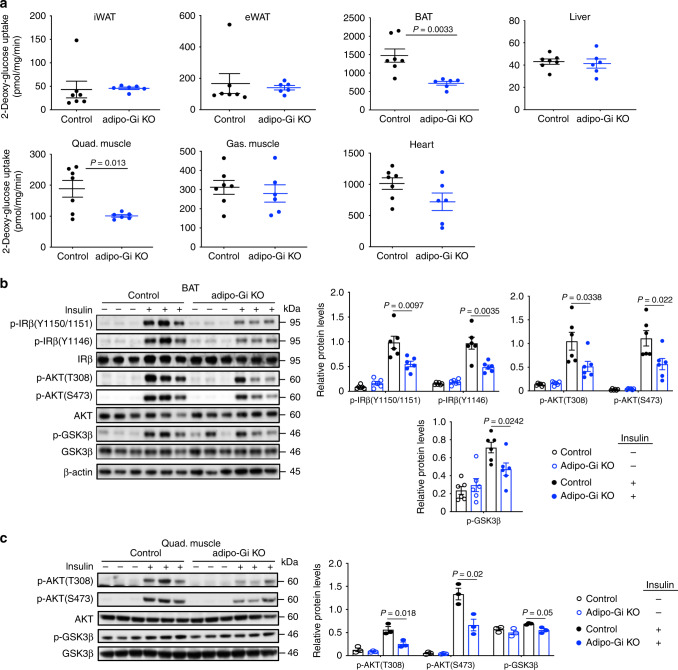
HFD adipo-Gi KO mice show impaired glucose uptake and insulin signaling in BAT and skeletal muscle. **a** In vivo 2-deoxy-glucose uptake studies. Control and adipo-Gi KO mice (males) maintained on a HFD for 8 weeks received an i.p. insulin injection (0.75 U/kg). Insulin-stimulated glucose uptake into inguinal white adipose tissue (iWAT), epididymal WAT (eWAT), brown adipose tissue (BAT), quadriceps muscle (Quad.), gastrocnemius muscle (Gas.), liver, and heart was studied using the 2-deoxy-glucose method (control, *n* = 7; adipo-Gi KO, *n* = 6). **b**, **c** Western blotting analysis of insulin signaling in BAT (**b**) (*n* = 6 per group) and quadriceps muscle (**c**) (*n* = 3 per group). HFD control and adipo-Gi KO mice were injected i.v. with either saline or insulin (5 U). Tissues were collected 5 min later and processed for immunoblotting studies. Data are given as means ± s.e.m. *P* values are indicated in the different panels (**a**: two-tailed Student’s *t* test; **b**, **c**: two-way ANOVA followed by Bonferroni’s post hoc test). Source data are provided as a Source data file.

The clamp studies indicated that insulin-stimulated glucose uptake was reduced in skeletal muscle and BAT of HFD adipo-Gi KO mice (Fig. 3f). To further confirm this finding, we subjected HFD adipo-Gi KO mice and control littermates to in vivo ^14^C-2-deoxy-glucose (^14^C-2-DG) uptake assays (Fig. 6a). Mice were fasted overnight and then injected with 0.75 U/kg insulin and a trace amount of ^14^C-2-DG. Sixty min later, the mice were sacrificed and peripheral tissues (iWAT, eWAT, BAT, various skeletal muscle tissues, heart, and liver) were collected. Subsequently, tissue ^14^C-2-DG accumulation was determined as a measure of tissue glucose uptake. In agreement with the clamp data, we found that insulin-stimulated glucose uptake was significantly decreased in BAT and skeletal muscle (quadriceps muscle) of HFD adipo-Gi KO mice (Fig. 6a).

To further confirm that insulin signaling was impaired in BAT and quadriceps muscle of HFD adipo-Gi KO mice, we injected HFD adipo-Gi KO mice and control littermates with insulin (5 U/mouse i.v.) or saline and collected BAT and skeletal muscle tissue 5 min later. Western blotting studies showed that insulin receptor signaling was significantly reduced in both tissues prepared from HFD adipo-Gi KO mice (Fig. 6b, c). We obtained similar data when we studied insulin receptor signaling in iWAT from HFD adipo-Gi KO and control mice (Supplementary Fig. 10). Despite this latter finding, insulin-stimulated ^14^C-2-DG uptake was not significantly affected in iWAT lacking functional G_i_ (Fig. 6a), perhaps due to considerable data scatter in the in vivo 2-DG uptake studies.

### Increased PTP1B activity impairs adipocyte insulin signaling

**Fig. 7:**
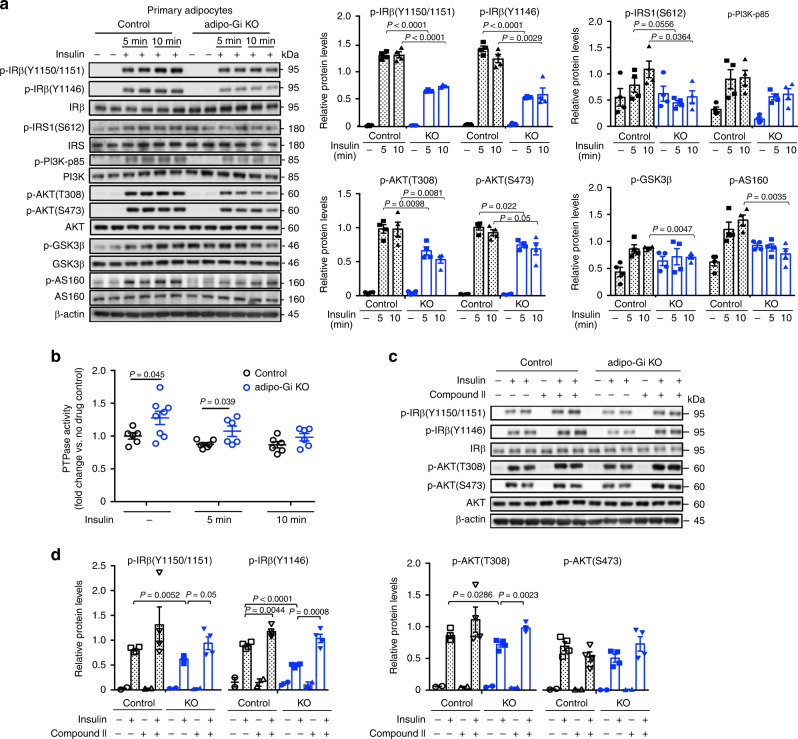
Lack of adipocyte G_i_ signaling impairs insulin signaling in adipocytes via enhanced PTPase activity. **a** Western blotting analysis of insulin signaling in primary adipocytes (iWAT) from adipo-Gi KO and control mice (males) treated with saline or 10 nM insulin for 5 or 10 min (*n* = 4 per group). **b** PTPase activity of primary adipocytes from adipo-Gi KO and control mice treated with either saline (−) (control, *n* = 6; adipo-Gi KO, *n* = 8) or 10 nM insulin for 5 or 10 min (control, *n* = 6; adipo-Gi KO, *n* = 6). **c** Immunoblots showing insulin-stimulated phosphorylation of IRβ-Y1150/1151, IRβ-Y1146, AKT-T308, and AKT-S473 in primary adipocytes from adipo-Gi KO and control mice. Adipocytes were treated with insulin (10 nM) for 5 min, either in the absence or presence of 500 nM Compound II (PTP1B inhibitor) (*n* = 4 per group). **d** Quantification of Western blotting data shown in (**c**). Data are presented as means ± s.e.m. *P* values are indicated in the different panels (**a**, **d**: two-way ANOVA followed by Bonferroni’s post hoc test; **b**: two-tailed Student’s *t* test). Source data are provided as a Source data file.

Previous work suggests that insulin receptor signaling can be modulated by the activity of heterotrimeric G proteins^32–34^. To investigate the effect of G_i_ signaling on insulin receptor signaling in adipocytes, we stimulated differentiated primary adipocytes from iWAT of adipo-Gi KO and control mice with insulin (10 nM) for 5 and 10 min. Western blotting studies showed that insulin receptor signaling, including insulin receptor auto-phosphorylation, was significantly impaired in the absence of functional G_i_ proteins (Fig. 7a). Protein tyrosine-phosphatase (PTPase) is known to inhibit insulin receptor auto-phosphorylation^35^. Strikingly, we found that PTPase activity was significantly increased in adipocytes lacking functional G_i_ proteins (Fig. 7b). Since PTPase 1B (PTP1B) is the most important PTPase expressed by adipocytes^36^, we hypothesized that a specific PTP1B inhibitor (compound II)^37,38^ would improve impaired insulin signaling caused by deficient adipocyte G_i_ signaling. Consistent with this notion, treatment of adipo-Gi KO adipocytes with compound II (500 nM) restored “control-like” insulin receptor signaling (Fig. 7c, d). These results indicate that intact G_i_ signaling is required for proper insulin receptor signaling in adipocytes.

### Enhanced cAMP/PKA signaling increases PTP1B activity

**Fig. 8:**
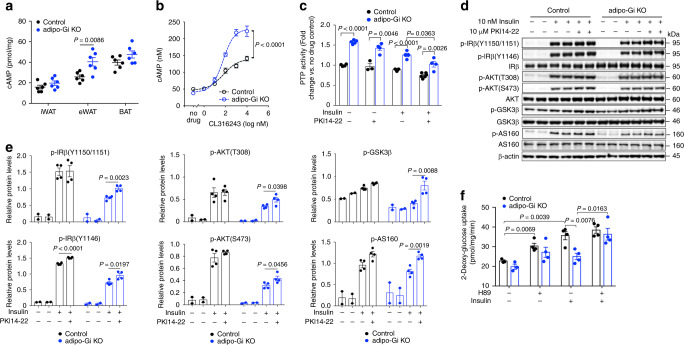
Deficient adipocyte G_i_ function enhances cAMP/PKA signaling and PTPase activity, causing impaired insulin signaling. **a** cAMP levels in adipose tissues from HFD adipo-Gi KO and control mice (males; *n* = 6 per group). **b** CL316243-induced stimulation of cAMP accumulation in primary adipocytes (iWAT) from control and adipo-Gi KO mice (*n* = 8 per group). **c** PTPase activity in primary adipocytes from adipo-Gi KO and control mice treated with either saline or insulin (10 nM) for 5 or 10 min, either in the absence or presence of PKA inhibitor (10 μM PKI14-22) (control, *n* = 3; adipo-Gi KO, *n* = 4). **d** Immunoblots showing the effect of PKA inhibition on insulin-stimulated phosphorylation of IRβ-Y1150/1151, IRβ-Y1146, AKT-T308, AKT-S473, GSK3β, and AS160 in primary adipocytes prepared from iWAT of adipo-Gi KO and control mice (diet: regular chow) were treated with 10 nM insulin for 5 or 10 min, either in the absence or presence of PKI14-22 (10 μM) (n = 4 per group). **e** Quantification of Western blotting data shown in (**d**). **f** Insulin-stimulated 2-deoxyglucose uptake by primary adipocytes. Primary adipocytes prepared from iWAT of adipo-Gi KO and control mice were treated with 10 nM insulin for 5 min, either in the absence or presence of H89 (10 μM) (*n* = 4 per group). Data are presented as means ± s.e.m. *P* values are indicated in the different panels (two-way ANOVA followed by Bonferroni’s post hoc test). PKI14-22, myristoylated PKI [14–22] amide. Source data are provided as a Source data file.

Previous studies have shown that stimulation of the cAMP/PKA pathway can enhance the activity of PTP1B^39,40^. We therefore hypothesized that the increase in PTPase activity displayed by adipo-Gi KO adipocytes might be due to the lack of the inhibitory effect of G_i_ signaling on cAMP formation. To address this issue, we measured cAMP levels in adipose tissues from HFD control and adipo-Gi KO mice. We found that cAMP levels were significantly increased in eWAT from adipo-Gi KO mice (Fig. 8a; iWAT and BAT showed a trend toward increased cAMP levels).

We next treated primary adipocytes from adipo-Gi KO and control mice with increasing concentrations of CL316243, a selective β3-adrenergic receptor agonist (the β3-receptor is the most abundant β-adrenergic receptor subtype in mouse adipocytes). CL316243 stimulated the production of cAMP in adipocytes from both groups of mice (Fig. 8b). However, this response was significantly more robust in adipocytes deficient in adipocyte G_i_ signaling (Fig. 8b). These data suggest that the increase in PTPase activity displayed by the adipo-Gi KO adipocytes is caused by enhanced cAMP/PKA signaling.

To provide additional support for this concept, we examined whether treatment of adipo-Gi KO adipocytes with a selective PKA inhibitor (myristoylated PKI [14–22] amide; abbreviated as PKI14–22 in the present study) was able to lower the increase in PTP1B activity characteristic for the mutant adipocytes. We found that PKI14–22 (10 μM) treatment caused a significant decrease in PTPase activity in adipo-Gi KO adipocytes prepared from iWAT of HFD mice under both basal conditions (no drug) and after stimulation with insulin (5 min; Fig. 8c). In addition, Western blotting studies indicated that the impairments in insulin receptor signaling found with adipocytes lacking functional G_i_ proteins could be significantly improved by PKI14–22 (10 μM)-mediated inhibition of PKA (Fig. 8d, e). We obtained similar data with H89 (10 μM), a widely used PKA inhibitor (Supplementary Fig. 11). In sum, these data strongly suggest that G_i_-mediated suppression of cAMP production and PKA activation is required for efficient insulin receptor signaling in adipocytes.

We next examined whether inhibition of PKA was able to reverse the deficits in insulin-stimulated glucose uptake displayed by the adipo-Gi KO adipocytes prepared from iWAT (Fig. 8d, e). We studied glucose uptake into primary adipocytes from control and adipo-Gi KO mice using the 2-deoxy-glucose method. While glucose uptake was greatly impaired when adipo-Gi KO adipocytes were treated with insulin alone (as compared to control adipocytes), co-treatment of insulin with H89 (10 μM) fully restored efficient glucose uptake in the adipo-Gi KO adipocytes (Fig. 8f).

While primary Gi KO adipocytes prepared from iWAT displayed impaired insulin-stimulated glucose uptake (Fig. 8f), the lack of adipocyte G_i_ signaling had no significant effect on glucose uptake in iWAT and eWAT in the hyperinsulinemic-euglycemic clamp study (Fig. 3f). Possible reasons for this discrepancy are that glucose uptake into white adipose tissue is very small in the clamp study and that many local and circulating factors modulate adipose tissue function in the vivo studies.

### Treatment of HFD adipo-Gi KO mice with a PTP1B inhibitor

To examine whether pharmacological inhibition of PTP1B was able to improve insulin resistance and glucose intolerance in HFD adipo-Gi KO mice, we carried out additional studies with a selective PTP1B inhibitor, CPT157633^41^. HFD control and adipo-Gi KO mice (age: 12 weeks, HFD during weeks 7–12) were fasted overnight for ~16 h (IGTT) or ~4 h (ITT), respectively. Mice were then injected with CPT157633 (5 mg/kg i.p.) or saline. Thirty min later, mice were subjected to glucose and insulin tolerance tests (IGTT and ITT, respectively). We found that treatment of HFD adipo-Gi KO mice with the PTP1B inhibitor restored control-like glucose tolerance and insulin sensitivity (Supplementary Fig. 12), indicating that enhanced PTP1B activity makes a major contribution to the in vivo metabolic deficits displayed by the HFD adipo-Gi KO mice.

### Adipocyte G_i_ signaling improves glucose homeostasis

To selectively activate adipocyte G_i_ signaling in vivo, we took advantage of the availability of a CNO-sensitive designer GPCR that selectivity couples to G proteins of the G_i_ family (official name: hM4Di; other names: G_i_ DREADD or simply GiD)^18,19^. To obtain mutant mice that express the GiD receptor selectively in adipocytes (adipo-GiD mice), we crossed *Rosa26-LSL-hM4Di* mice^42^ with *adipoq-Cre* mice^20^. *Rosa26-LSL-hM4Di* mice that lacked the *adipoq-Cre* transgene served as control animals in all studies.

**Fig. 9:**
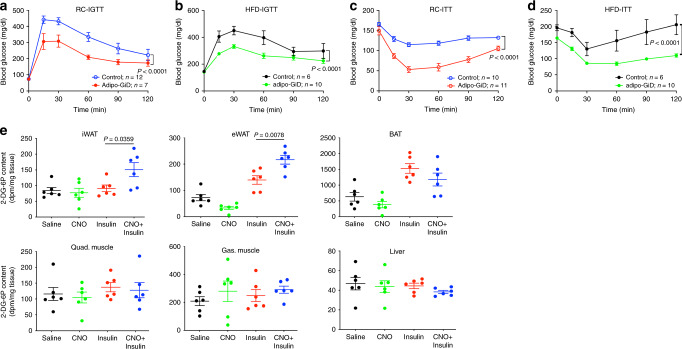
Activation of adipocyte G_i_ signaling improves glucose metabolism and insulin sensitivity. Adipo-GiD mice and control littermates (adult males) were subjected to a series of metabolic tests. **a**, **b** I.p. glucose tolerance test (IGTT) (RC, regular chow: 10 mg/kg CNO + 2 g/kg glucose; HFD: 10 mg/kg CNO + 1 g/kg glucose). **c**, **d** Insulin tolerance test (ITT) (RC, regular chow, 10 mg/kg CNO + 0.75 U/kg insulin, i.p.; HFD, 10 mg/kg CNO + 1 U/kg insulin, i.p.). **e** In vivo 2-deoxy-glucose uptake in iWAT, eWAT, BAT, quadriceps muscle (Quad.), gastrocnemius muscle (Gas.), and liver of 8-week-old adipo-GiD mice maintained on RC. Mice that had been fasted overnight were injected i.p with saline, 10 mg/kg CNO, 0.75 U/kg insulin, or 10 mg/kg CNO + 0.75 U/kg insulin (*n* = 6 per group). Data are given as means ± s.e.m. *P* values are indicated in the different panels (two**-**way ANOVA followed by Bonferroni’s post hoc test). Source data are provided as a Source data file.

After acute CNO injection (10 mg/kg i.p), plasma FFA, glycerol, and triglyceride levels were markedly decreased in RC and HFD adipo-GiD mice but not in control littermates (Supplementary Fig. 13a, b), indicating that CNO could specifically activate adipocyte G_i_ signaling. CNO treatment (10 mg/kg i.p) of adipo-GiD mice also resulted in significantly decreased plasma insulin levels (Supplementary Fig. 13a, b), probably caused by an increase in insulin sensitivity secondary to reduced plasma FFA levels. Strikingly, CNO (10 mg/kg i.p)-induced activation of adipocyte G_i_ signaling in adipo-GiD mice resulted in pronounced improvements in glucose tolerance and insulin sensitivity, independent of the diet that the mice consumed (RC or HFD; Fig. 9a–d). In vivo ^14^C-2-DG uptake assays demonstrated that CNO (10 mg/kg i.p.) mediated activation of adipocyte G_i_ signaling significantly enhanced insulin-induced glucose uptake into adipose tissues (iWAT and eWAT; Fig. 9e). This effect was not observed with CNO-treated control mice, indicating that it was dependent on activation of the G_i_-coupled designer receptor (Supplementary Fig. 14).

Caron et al.^13^ recently reported that CNO treatment (1 mg/kg i.p.) of adipo-GiD mice led to a modest but significant increase in plasma leptin levels 2 h following CNO injection. Somewhat surprisingly, we found that CNO treatment (1 or 10 mg/kg i.p.) of adipo-GiD mice or control littermates did not lead to any significant changes in plasma leptin levels throughout the 2 h observation period (Supplementary Fig. 15a, d). However, CNO treatment (1 or 10 mg/kg i.p.) of the same set of animals resulted in significant decreases in plasma FFA levels in adipo-GiD mice, but not in control littermates (Supplementary Fig. 15b, e). CNO administration (1 or 10 mg/kg i.p.) caused only mild increases in blood glucose levels that did not differ significantly between adipo-GiD and control mice (Supplementary Fig. 15c, f). Most likely, these effects resulted from glucose mobilization due to the injection stress.

**Fig. 10:**
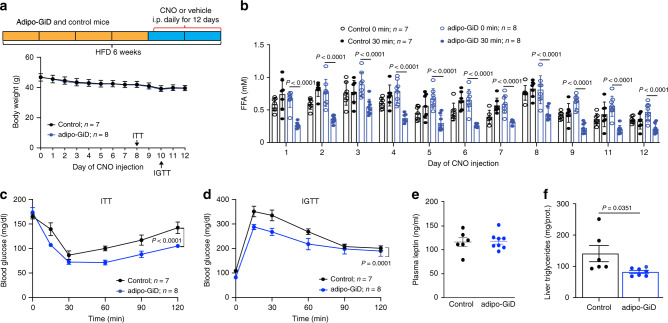
Chronic activation of adipocyte G_i_ signaling improves glucose homeostasis. **a** Scheme of the experimental design used (top). Control and adipo-GiD mice (males) consumed a HFD for 6 weeks. During the last 12 days, mice received daily i.p. injections of CNO (10 mg/kg). **b** Plasma free fatty acid (FFA) levels during chronic CNO treatment of HFD adipo-GiD mice and control littermates. Plasma FFA levels were determined daily before and 30 min after CNO treatment. **c** Insulin tolerance test (ITT) after chronic CNO treatment for 8 days (0.75 U/kg insulin, i.p.). **d** I.p. glucose tolerance test (IGTT) after chronic CNO treatment for 10 days (1 g/kg glucose). **e** Plasma leptin levels of HFD adipo-GiD and control mice after CNO treatment for 12 days (control, *n* = 6; adipo-GiD, *n* = 8). **f** Liver triglyceride content of HFD adipo-GiD and control mice after CNO treatment for 12 days (control, *n* = 6; adipo-GiD, *n* = 7). Data are given as means ± s.e.m. *P* values are indicated in the different panels (**b**–**d**: two-way ANOVA followed by Bonferroni’s post hoc test; **f**: two-tailed Student’s *t* test). Source data are provided as a Source data file.

To examine whether chronic activation of adipocyte G_i_ signaling was able to improve glucose homeostasis in vivo, we maintained control and adipo-GiD mice on HFD for 6 weeks. During the last 12 days of HFD feeding, the mice received daily i.p. injections of either CNO (10 mg/kg) or saline (control) (Fig. 10a). CNO treatment had essentially no effect on body weight in both control and adipo-GiD mice (Fig. 10a). However, CNO-treated adipo-GiD mice showed significantly reduced plasma FFA levels throughout the entire CNO administration period, as compared to the three control groups (Fig. 10b). Strikingly, during chronic CNO treatment, adipo-GiD mice showed improved insulin sensitivity and glucose tolerance, as compared to CNO-treated control littermates (Fig. 10c, d). Plasma leptin levels were not significantly different at the end of the 12-day CNO treatment period (Fig. 10e), in agreement with the acute CNO injection data (Supplementary Fig. 15a, d).

Liver triglyceride content was significantly decreased in adipo-GiD mice chronically treated with CNO (Fig. 10f). These data indicate that activation of adipocyte G_i_ signaling can overcome the metabolic deficits associated with the consumption of a HFD.

### G_i_-coupled receptors endogenously expressed by adipocytes

The metabolic phenotypes displayed by the CNO-treated adipo-GiD mice suggested that targeting G_i_-coupled receptors endogenously expressed by adipocytes might prove beneficial to treat impairments in glucose homeostasis in T2D. To identify such receptors, we subjected RNA prepared from isolated mouse adipocytes (iWAT and eWAT) and BAT tissue to RNA-seq analysis. This analysis demonstrated that mouse adipocytes/BAT tissue express a large number of GPCRs that are selectively coupled to G_i_-type G proteins, including many chemokine receptor subtypes, the succinate receptor (succinate receptor 1), the CB_1_ cannabinoid receptor, the HCA receptors 1 and 2, and several orphan receptors (Supplementary Table 1). Interestingly, we observed that the expression of many of these receptors were upregulated in HFD mice.

## Discussion

We here report that mice lacking functional G_i_ proteins selectively in adipocytes (adipo-Gi KO mice) showed pronounced impairments in glucose homeostasis when maintained on an obesogenic diet (HFD). Hyperinsulinemic-euglycemic clamp studies showed that GIR was dramatically decreased in HFD adipo-Gi KO mice (Fig. 3b), confirming that the lack of adipocyte G_i_ signaling causes severe insulin resistance. Additional experiments showed that the profound insulin resistance displayed by HFD adipo-Gi KO mice is due to impaired insulin action on multiple peripheral tissues or organs, including liver, skeletal muscle, and adipose tissue (BAT), most likely due to enhanced breakdown of adipocyte triglycerides (see below).

Interestingly, the mRNA expression levels of several pro-inflammatory cytokines were significantly elevated in adipose tissues from HFD adipo-Gi KO mice (Fig. 5b). Moreover, adipocytes from HFD adipo-Gi KO mice (iWAT and eWAT) mice were extensively infiltrated by mononuclear inflammatory cells/macrophages (Fig. 5b, c; Supplementary Fig. 8), indicating that the lack of adipocyte G_i_ signaling causes adipose tissue inflammation.

In agreement with the inflammatory processes caused by the lack of adipocyte G_i_ signaling, Western blotting studies showed that insulin receptor signaling, including insulin receptor auto-phosphorylation, was significantly impaired in the absence of adipocyte G_i_ signaling (Fig. 7a). We also found that PTPase activity, which is known to inhibit insulin receptor auto-phosphorylation^35^, was significantly increased in adipocytes lacking functional G_i_ proteins (Fig. 7b). Strikingly, treatment of adipo-Gi KO adipocytes with compound II, a specific PTP1B inhibitor, fully restored efficient insulin receptor signaling (Fig. 7c), indicating that intact G_i_ signaling is required for proper insulin-mediated regulation of adipocyte metabolism.

It has been reported that stimulation of the cAMP/PKA pathway leads to the activation of PTP1B^39,40^. In agreement with this concept, we obtained data strongly suggesting that G_i_-mediated suppression of cAMP-mediated PKA activation is required for efficient insulin receptor signaling in adipocytes (Fig. 8a–e).

A key question is how the metabolic and functional deficits displayed by adipose tissue deficient in adipocyte G_i_ signaling can cause severe peripheral insulin resistance. We found that plasma FFA levels were significantly elevated in adipo-Gi KO mice (Fig. 1f and Supplementary Fig. 2e), most likely due to an increase in the rate of lipolysis in adipose tissue (Fig. 2a, b). Many studies have shown that increased plasma FFA levels can trigger insulin resistance and inflammation in the major insulin target tissues, thus linking obesity with insulin resistance and the development of T2D^25^. To study the contribution of elevated plasma FFA levels to the metabolic deficits displayed by the HFD adipo-Gi KO, we treated HFD adipo-Gi KO mice with the HSL inhibitor BAY 59–9435 (Bay)^26,27^ to lower plasma FFA levels to levels similar to those displayed by control mice (Fig. 4b). Strikingly, this manipulation greatly improved glucose tolerance and insulin sensitivity in the HFD adipo-Gi KO mice (Fig. 4c, d), restored efficient insulin signaling in skeletal muscle (quadriceps) and liver (Fig. 4f, i), and markedly reduced liver weight and hepatic triglyceride content (Fig. 4g, h). These data clearly indicate that the increase in plasma FFA levels caused by deficient adipocyte G_i_ signaling makes a key contribution to the metabolic deficits caused by the lack of adipocyte G_i_ function.

Interestingly, Bay treatment of HFD adipo-Gi KO mice also reduced elevated gene expression levels of inflammatory cytokines in iWAT and eWAT (Supplementary Fig. 9b, c), reduced elevated plasma levels of inflammatory cytokines (Supplementary Fig. 9a), greatly reduced increased macrophage infiltration of adipose tissues (Supplementary Fig. 8), and restored control-like morphology to adipose tissue of HFD adipo-Gi KO mice (Supplementary Fig. 9d). These findings suggest that the lack of adipocyte G_i_ signaling causes an increase in plasma FFA levels, which in turn trigger inflammatory processes that contribute to the metabolic deficits displayed by the HFD adipo-KO mice.

Based on these findings, we speculated that activation of adipocyte G_i_ signaling may cause beneficial metabolic effects. Indeed, CNO-induced activation of adipocyte G_i_ signaling in adipo-GiD mice caused striking improvements in glucose and insulin tolerance, independent of the diet that the mice consumed (Fig. 9a–d). These beneficial metabolic effects were accompanied by enhanced insulin-induced glucose uptake into adipose tissues (Fig. 9e). Moreover, chronic CNO treatment of adipo-GiD mice greatly improved the metabolic impairments associated with the consumption of an obesogenic HFD, including insulin resistance, reduced glucose tolerance, and hepatic steatosis (Fig. 10c, d, f). These CNO-dependent improvements were accompanied by a significant reduction in plasma FFA levels (Fig. 10b), suggesting that G_i_-mediated inhibition of lipolysis plays a key role in the beneficial metabolic profile displayed by the CNO-treated HFD adipo-GiD mice.

Recently, Caron et al.^13^ characterized an adipo-GiD mouse line that was generated by using the same strategy used in the present study. Interestingly, these authors showed that CNO treatment (1 mg/kg i.p.) of adipo-GiD mice led to a modest but significant increase in plasma leptin levels 2 hr following CNO injection. However, we found that CNO treatment (1 or 10 mg/kg i.p.) of adipo-GiD mice or control littermates did not lead to any significant changes in plasma leptin levels throughout the 2 h observation period (Supplementary Fig. 15a, d). A possible reason for these discrepant findings is that the mice used by Caron et al. were considerably younger than the ones used in the present study (age: 8–14-weeks vs. 32 weeks, respectively). Differences in environment, diet, etc. may also be contributing factors.

The metabolic phenotypes displayed by the CNO-treated adipo-GiD mice suggest that G_i_-coupled receptors that are endogenously expressed by adipocytes (see Supplementary Table 1) may represent drug targets for improving glucose homeostasis for therapeutic purposes (e.g. for the treatment of T2D). Our RNA-seq data revealed that the expression of many of these receptors are upregulated in HFD mice (Supplementary Table 1), a finding that could potentially be exploited therapeutically.

In conclusion, we found that adipocyte G_i_ signaling is essential for maintaining euglycemia and proper peripheral insulin sensitivity. Drug-mediated activation of adipocyte G_i_-coupled receptors may prove useful to improve impaired blood glucose homeostasis and insulin resistance in T2D.

## Methods

### Drugs

All drugs used are listed in Supplementary Table 2.

### Animals

To generate mice lacking functional G_i_ proteins selectively in adipocytes (“adipo-Gi KO mice”), we used *ROSA26-PTX*^*flox/flox*^ mice which contain the gene encoding the catalytic subunit of PTX (S1-PTX), a known G_i_ signaling inhibitor^21,43^. In this mouse strain, the S1-PTX coding sequence is preceded by a flox-stop sequence. In order to achieve selective expression of S1-PTX in adipocytes, we crossed *adipoq-Cre* mice^20^ (Jackson Laboratories, No. 010803; genetic background: C57BL/6J) with *ROSA26-PTX*^*flox/flox*^ mice. Throughout the text, we refer to the resulting *adipoq-Cre ROSA26-PTX*^*flox/+*^ as ‘adipo-Gi KO’ mice. *ROSA26-PTX*^*flox/+*^ mice lacking the *adipoq-Cre* allele served as control mice. All mice were maintained on a C57BL/6 background.

We also generated mutant mice expressing a CNO-sensitive G_i_ DREADD, also referred to as GiD or hM4Di^18^ (DREADD = designer receptor exclusively activated by a designer drug,) selectively in adipocytes (“adipo-GiD mice”). These mice were obtained by crossing *adipoq-Cre* mice^20^ with *Rosa26-LSL-hM4Di* mice^42^. *Rosa26-LSL-hM4Di* mice lacking the *adipoq-Cre* transgene served as control animals throughout the study (genetic background of control and adipo-GiD mice: C57BL/6).

Unless stated otherwise, adult male littermates were used for all experiments. Mice were housed under conditions of controlled temperature (23 °C) and illumination (12-h light/12-h dark cycle; light off at 6 p.m.). The animals had free access to water and food. Mice consumed either a standard chow (7022 NIH-07 diet, 15% kcal fat, energy density 3.1 kcal/g, Envigo Inc.) or a high-fat diet (HFD; F3282, 60% kcal fat, energy density 5.5 kcal/gm, Bioserv). Mice consumed the HFD for at least 8 weeks, unless stated otherwise. Mice were switched from the regular chow diet to the HFD when they were 6 weeks old.

All animal studies were carried out according to the US National Institutes of Health Guidelines for Animal Research and were approved by the NIDDK Institutional Animal Care and Use Committee.

### In vivo metabolic tests

In vivo metabolic tests were performed with male mice (mouse age: 8–20 weeks) using standard procedures. I.p. glucose tolerance tests (IGTT) were performed with mice that had been fasted overnight for ~16 h. Blood glucose concentrations were determined before and after injection of mice with an i.p. glucose bolus (1 or 2 g/kg, as indicated). Blood glucose levels were determined at defined post-injection time points by analyzing blood obtained from the tail vein with a portable glucometer (Glucometer Elite Sensor; Bayer). For insulin tolerance tests (ITT), mice were fasted for 4 h and then injected with human insulin (0.75 or 1 U/kg i.p.; Humulin, Eli Lilly). Blood glucose levels were monitored as described for the IGTT assay. Pyruvate tolerance tests (PTT) were carried out with mice that had been fasted for 16 h, followed by an i.p. injection of sodium pyruvate (2 g/kg)^43^ and monitoring of blood glucose levels. To study glucose-stimulated insulin secretion (GSIS), mice were fasted overnight and then injected i.p. with 1 or 2 g/kg of glucose, as indicated. Blood was collected at specific post-injection time points, and plasma insulin levels were detected using a mouse insulin ELISA kit (Crystal Chem Inc.), according to the manufacturer’s instructions.

Plasma glycerol, triglyceride, and FFA levels were determined using commercially available kits (Sigma-Aldrich and Fujifilm Wako Diagnostics). Plasma leptin and adiponectin levels were measured using ELISA kits (R&D Systems).

To study niacin-induced changes in plasma FFA levels, control and adipo-Gi KO mice were fasted overnight for ~12 h and then injected with niacin (100 mg/kg, i.p.). At defined post-injection time points (30 and 60 min), blood was collected from the tail vein, followed by the measurement of plasma FFA levels.

In order to lower plasma FFA levels, control and adipo-Gi KO mice were treated with Bay (BAY 59–9435, Pfizer), an inhibitor of hormone-sensitive lipase (HSL)^44^. Mice were given 30 mg/kg Bay or 0.5% methylcellulose (vehicle) via oral gavage once per day for two weeks.

### CNO treatment of adipo-GiD mice

Adipo-GiD mice and control littermates (males) were fasted for 4 h, followed by the i.p. injection of either 1 or 10 mg/kg CNO. The data shown in Supplementary Fig. 13 were obtained with mice (age: 8–12 weeks) that had been maintained on regular chow (RC) or a high-fat diet (HFD; mouse age: 16 weeks; mice were 6 weeks old when they were switched from RC to HFD). The data shown in Supplementary Fig. 15 were generated with 32-week-old RC mice. Blood was collected from the tail vein at 0, 30, 60 and 120 min after CNO injection. Plasma was obtained by centrifuging blood samples at ~12,000 × *g* for 10 min at 4 °C. Plasma FFA and leptin levels were determined using commercially available ELISA kits from Fujifilm Wako Diagnostics and R&D Systems, respectively. For chronic CNO treatment studies, adipo-GiD mice and control littermates were fed with HFD for 6 weeks (HFD feeding was initiated when the mice were 8 weeks old). During the last 12 days of HFD feeding, mice received single daily injections of CNO (10 mg/kg i.p.).

### Hyperinsulinemic-euglycemic clamp

All procedures required for the hyperinsulinemic-euglycemic clamp were approved by the Vanderbilt University Animal Care and Use Committee. Catheters were implanted into a carotid artery and a jugular vein of mice for sampling and infusions five days before the study^45^. Insulin clamps were performed on mice fasted for 5 h using a modification of the procedure reported by Ayala et al.^46^. [3–^3^H]-glucose was primed (1.5 μCi) and continuously infused for a 90 min equilibration and basal sampling period (0.075 μCi/min). [3-^3^H]-glucose was mixed with the non-radioactive glucose infusate (specific activity of the infusate: 0.5 μCi/mg) during the 2 h clamp period. Arterial glucose was clamped using a variable rate of glucose (plus [3–^3^H]-glucose tracer) infusion, which was adjusted based on blood glucose measurements at 10 min intervals. By mixing radioactive glucose with the non-radioactive glucose infused during a clamp, deviations in arterial glucose specific activity are minimized and steady state conditions are achieved. The calculation of glucose kinetics is therefore more robust^47^. Baseline blood or plasma variables were calculated as the mean of values obtained in blood samples collected at −15 and −5 min. At time “0”, the insulin infusion (4 mU/kg/min) was started and continued for 120 min. Mice received heparinized, saline-washed erythrocytes from donors at 5 μl/min to prevent a fall in hematocrit. Blood was taken during the 80–120 min period for the determination of [3-^3^H]-glucose. Clamp insulin was determined at 100 and 120 min. At 120 min, 13 µCi of 2[^14^C]-deoxyglucose ([^14^C]2DG) was administered as an intravenous bolus. Blood was taken 2–25 min later for the determination of [^14^C]2DG. At the end of the experiment, mice were anesthetized, and tissues were freeze-clamped for further analysis. Plasma insulin was determined with a RIA kit. Radioactivity of [3-^3^H]-glucose and [^14^C]2DG in plasma samples, and [^14^C]2DG-6-phosphate in tissue samples were determined by liquid scintillation counting. Glucose appearance (Ra) and disappearance (Rd) rates were determined using steady-state equations^48^. Endogenous glucose appearance (endo-Ra) was determined by subtracting the glucose infusion rate (GIR) from the total Ra.

### Body composition analysis

The lean/fat mass composition of mice was measured using the 3-in-1 Echo MRI Composition Analyzer (Echo Medical System).

### Indirect calorimetry

Indirect calorimetry and energy expenditure measurements were carried out using Oxymax-CLAMS (Columbus Instruments) chambers^49,50^. Mice that had been maintained on HFD for 12 weeks were acclimatized to the chambers for 2 days at 22 °C. On day 3, energy expenditure and related parameters including food intake and locomotor activity were studied at the same temperature (22 °C). On day 4, then temperature of the chambers was raised to 30 °C (thermoneutrality), and the same metabolic parameters were recorded as on day 3. For each mouse, food intake, O_2_ consumption, CO_2_ production, and ambulatory activity (infrared beam breaks) were determined every 13 min. Total energy expenditure (TEE) and respiratory exchange ratio (RER) were calculated based on O_2_ consumption and CO_2_ production.

### Measurement of food intake

Adipo-Gi KO mice and control littermates were housed individually and maintained on a HFD for 6 weeks. During the last 2 weeks of HFD feeding, mice received BAY 59–9435 (30 mg/kg) or vehicle (0.5% methylcellulose) via oral gavage once per day. Food intake was measured daily during the Bay treatment period.

### Measurement of mouse plasma adipokine and cytokine levels

Mouse blood was obtained from the tail vein and collected in K_2_-EDTA-containing tubes (RAM Scientific). Blood samples were quickly centrifuged at 4 °C to obtain plasma. Plasma adipokine and cytokine levels were measured using the Bio-Plex Multiplex Immunoassay System (Bio-Rad), following the manufacturer’s instructions. Adipokine/cytokine concentrations were determined by the Luminex Milliplex Analyzer (Luminex), as indicated by the manufacturer.

### Isolation of mature adipocytes and SVF cells from adipose tissue

iWAT from 8–12-week-old male mice (diet: regular chow) was isolated and digested at 37 °C for 30–45 min in Krebs-Ringer-Hepes-bicarbonate buffer (KRH buffer, 1.2 M NaCl, 40 mM KH_2_PO_4_, 20 mM MgSO_4_, 10 mM CaCl_2_, 100 mM NaHCO_3_, and 300 mM HEPES) containing 3.3 mg/ml collagenase I (Sigma-Aldrich). Once the tissue was fully digested, 10 ml of KRH buffer was added to prevent further collagenase activity. Cells were then filtered through a 250 μm cell strainer. Ten min later, the floating top layer containing mature adipocytes was collected. Mature adipocytes were washed twice with KRH buffer containing 5 mM EDTA. After collection of the mature adipocytes, the digested tissue suspension was filtered through a 100 μm cell strainer, followed by centrifugation at 400 g for 5 min at room temperature. The cell pellet was re-suspended in Dulbecco’s PBS (dPBS) and filtered through a 40 μm cell strainer. The cell suspension was centrifuged again as described above to obtain a pellet containing the stromal vascular fraction (SVF) cells.

### Preparation and differentiation of primary iWAT adipocytes

iWAT from 8–12-week-old male mice (diet: regular chow) was collected and minced into small pieces, followed by digestion at 37 ^o^C for 30–45 min in KRH buffer. Following complete tissue digestion, cell suspensions were diluted with 10–15 ml KRH buffer and filtered through a 100 μm cell strainer, followed by centrifugation at 300 × *g* for 5 min at room temperature. The fat layer was discarded, and the cell pellet was re-suspended in Dulbecco’s PBS (DPBS) and filtered through a 40 μm cell strainer. The cell suspension was re-centrifuged to obtain a pellet of the stromal vascular fraction (SVF). The SVF pellet was re-suspended in DMEM containing 10% bovine calf serum (BCS) and 1% pen-strep. The SVF cells were plated in collagen I-coated-6/12-well plates. Once the cells had reached confluency, they were differentiated into mature iWAT adipocytes by a 48 hr treatment with differentiation medium containing 0.5 μM insulin, 250 μM 3-isobutyl-1-methylxanthine (IBMX), 2 μM troglitazone, 0.5 μM dexamethasone, and 60 μM indomethacin. The medium was then removed, and cells were incubated with DMEM containing 10% BCS, 1% pen-strep, and 0.5 μM insulin for another 48 hr. After completion of the differentiation process, mature adipocytes were used for further studies.

### Real-time qRT-PCR gene expression analysis

Mouse tissues or mature adipocytes isolated from iWAT of control and adipo-Gi KO mice were collected and immediately frozen. Total mRNA was extracted and purified by using the RNeasy mini kit combined with the RNase-free DNase set from Qiagen, following the manufacturer’s protocol. cDNA was synthesized using SuperScript™ III First-Strand Synthesis SuperMix (Invitrogen). Real-time qPCR was performed using both the SYBR green and TaqMan methods (Applied Biosystems). TaqMan primer/probe sets for real-time PCR were designed using Primer Express software (Applied Biosystems) and were purchased from Integrated DNA Technologies. RNA expression data were normalized relative to the expression of β-actin or 18S rRNA. A complete list of PCR primers and Taqman probes used is provided in Supplementary Table 3.

### Western blotting studies

To monitor insulin signaling in different tissues, mice were deeply anesthetized via isoflurane inhalation (Baxter Healthcare Corporation), followed by the opening of the abdominal cavity. In the next step, 5 units of human insulin (Humulin, Eli Lilly) diluted in 100 μl 0.9% saline were injected into the inferior vena cava. Sham injections were performed with 100 μl of 0.9% saline. Five min after injections, tissues were harvested and snap-frozen in liquid nitrogen.

Tissue samples were homogenized in RIPA buffer (for liver and skeletal muscle) or adipocyte lysis buffer (50 mM Tris, pH 7.4, 500 mM NaCl, 1% NP40, 20% glycerol, 5 mM EDTA, and 1 mM phenylmethylsulphonyl fluoride, supplemented with complete EDTA-free protease inhibitor cocktail and phosphatase inhibitors cocktail from Roche). Tissue lysates were centrifuged at 16,000 × *g* for 10 min at 4 ^o^C, and the supernatants were aliquoted and used for subsequent analysis^51^. Protein concentrations were determined using a bicinchoninic acid assay kit (Pierce). Protein lysates were denatured at 70 ^o^C for 10 min in NuPAGE LDS sample buffer (Thermo Fisher Scientific) and then separated by 4–12% SDS-PAGE (Invitrogen) and transferred to nitrocellulose membranes (Bio-Rad). Membranes were incubated overnight with primary antibody in 5% w/v BSA, 1x TBS, and 0.1% Tween 20 at 4 °C, followed by incubation with anti-rabbit or anti-mouse IgG HRP-linked secondary antibodies. Specific protein bands were visualized using the Azure Imager C600 (Azure Biosystems) and SuperSignal West Pico Chemiluminescent Substrate (Pierce). The list of primary antibodies used is provided in Supplementary Table 2.

To monitor insulin receptor signaling via Western blotting in mature adipocytes, primary adipocytes were prepared from mouse iWAT and differentiated into mature adipocytes. Subsequently, differentiated adipocytes were serum-starved for 4 hr, followed by treatment with 10 nM insulin for 5 or 10 min.

To check for S1-PTX protein expression in mature adipocytes and cells of the stromal vascular fraction (SVF), mature adipocytes and SVF cells were isolated from iWAT of control and adipo-Gi KO mice maintained on regular chow.

### Liver triglyceride content

Mouse livers were homogenized in PBS. Total lipid was extracted from the homogenates with chloroform/methanol (2:1). An aliquot of the organic phase was collected, dried overnight, and re-suspended in 1% Triton X-100 ethanol. Hepatic triglyceride content was determined using a commercially available kit (Sigma Aldrich). Protein concentrations were determined by employing a bicinchoninic acid assay kit (Pierce). Liver triglyceride content was expressed as mg triglycerides/g protein.

### cAMP assay

Differentiated primary mature adipocytes from iWAT of control and adipo-Gi KO mice (diet: regular chow) were grown in collagen I-coated 12-well plates. Cells were incubated with or without 100 ng/ml PTX for 16 h in complete medium (DMEM containing 10% bovine calf serum (BCS) and 1% pen-strep). On the day of the cAMP assay, adipocytes were washed twice with DPBS and then starved in serum-free DMEM for 1 h. Both control and adipo-Gi KO adipocytes were pretreated with niacin (10 μM) or left untreated for 1 h. Subsequently, adipocytes were incubated at in DMEM containing 100 μM IBMX with increasing concentrations of CL316243 at 37 ^o^C for 1 h. Cells were lysed with 0.05 M HCl, and intracellular cAMP levels were determined using a FRET-based cAMP detection kit (Cisbio Bioassays)^52^. To determine the cAMP content of adipose tissues, tissue samples were homogenized according to the manufacturer’s protocol. The lysates were centrifuged at 15,000 × *g* for 15 min at 4 ^o^C, and cAMP levels were determined in the supernatant using a cAMP ELISA kit (Cayman Chemical).

### PTPase activity assay

Differentiated primary mature adipocytes from iWAT of control and mutant mice (diet: regular chow) were washed 3 times with PBS and then incubated for 4 hr in serum-free DMEM. Adipocytes were pre-treated with 10 μM of myristoylated PKI [14–22] amid (PKI14–22; selective PKA inhibitor) or left untreated for 1 h, followed by a 5 min incubation with 10 nM insulin. PTPase activity was determined by using the Protein Tyrosine Phosphatase Activity Assay Kit (Fluorometric) from BioVision. Adipocytes were homogenized in PTPase assay buffer containing 100 mM DTT (50:1 ratio; 20 μl of 100 mM DTT stock solution per 1 ml PTPase assay buffer). Tissue/cell homogenates were centrifuged at 10,000 × *g* at 4 °C for 15 min, followed by the collection of supernatants. Assays were performed with 20 μl of supernatant in the absence or presence of 200 μM suramin, a PTPase inhibitor. The enzymatic reaction was started by adding 20 μl of 5 x PTPase substrate solution. Fluorescence at Ex/Em = 368/460 nm was measured immediately in kinetic mode for 45 min at 25 °C (SpectraMax M5, Molecular Devices). Specific PTPase activity was calculated based on the amounts of metabolite produced and protein present in the sample wells.

### In vivo metabolic studies after treatment of mice with a PTP1B inhibitor

HFD control and adipo-Gi KO mice (age: 12 weeks; HFD during weeks 7–12) were fasted overnight for ~16 h (IGTT) or 4 h (ITT), respectively. Mice were then injected with CPT157633 (5 mg/kg i.p.), a selective PTP1B inhibitor^41^, or saline. Thirty min later, glucose and insulin tolerance tests were carried out as described under “In vivo metabolic tests”.

### ^3^H-2-deoxy-glucose uptake assay in adipocytes

Mature primary mouse adipocytes were serum-starved for 2–3 h in 12-well plates and then rinsed with HBS buffer solution of the following composition (in mM): 20 HEPES [pH 7.4], 2.5 MgSO_4_, 1 CaCl_2_, 140 NaCl, and 5 KCl. Adipocytes were preincubated with or without 10 μM H89 for 1 hr and then incubated with 10 nM insulin for 5 min at 37 ^o^C, followed by three washes with HBS buffer. Subsequently, cells were incubated for 5 min at 37 °C in transport solution (HBS buffer supplemented with 0.5 μCi/ml ^3^H-2-deoxy-glucose (PerkinElmer) and 100 mM 2-deoxy-d-glucose (Sigma)). To correct for non-specific glucose uptake, 10 μM cytochalasin B (Sigma) was added to the transport solution. Glucose uptake was terminated by the addition of ice-cold stop solution (0.9% NaCl solution containing 25 mM glucose). After three washes with ice-cold stop solution, cells were lysed in 0.05 N NaOH. Cell lysates were mixed with 7 ml of Ecoscint A scintillation fluid (National Diagnostics), and radioactivity was measured with a PerkinElmer liquid scintillation counter. Protein concentrations were determined by using the Bio-Rad Bradford assay kit. Results were expressed as uptake of ^3^H-2-deoxy-glucose (in pmoles) per mg of protein (pmoles/mg/min).

### ^14^C-2-deoxy-glucose uptake in mice

Mice were fasted overnight for ~16 h and then injected i.p. with either saline, 0.75 U/kg human insulin, 10 mg/kg CNO, or a mixture of 10 mg/kg CNO and 0.75 U/kg human insulin (adipo-GiD mice only) and 10 μCi of ^14^C-2-deoxy-glucose (^14^C-2-DG). Forty-five minutes after injections, the mice were euthanized, and tissues were collected. Tissue samples were weighed and homogenized, and radioactivity was measured and counted^53^.

### Lipolysis assay

Differentiated mature adipocytes prepared from iWAT of control and mutant mice (diet: regular chow) were used for lipolysis assays. In a subset of experiments, adipocytes were treated with PTX (100 ng/ml) for 24 h or left untreated. On the day of the assay, adipocytes were washed 3 times with PBS and then incubated for 3–4 h in serum-free DMEM. Subsequently, cells were incubated in KRB buffer (composition in mM: 135 NaCl, 5 KCl, 1 MgSO_4_, 0.4 K_2_HPO_4_, 20 HEPES, 1 CaCl_2_, 5 glucose, and 4% BSA, PH = 7.4) in the presence of various drugs (1 nM CL316243, 1 μM isoproterenol, 10 μM CNO, and/or 10 μM niacin), as indicated in the text (note that CL316243 and isoproterenol were used to simulate lipolysis). Incubations were carried out at 37 °C for 2 h. Subsequently, cell culture plates were placed on ice to terminate the reaction. Media were collected for the measurement of glycerol by using a glycerol assay kit (Sigma). Cells were washed several times with ice-cold PBS to remove BSA and then scraped into cell lysis buffer for the determination of protein concentrations. Data were expressed as amount of glycerol released per mg or μg of protein.

### Histology

Adipose and liver tissues were fixed in 4% paraformaldehyde for 24 h and embedded in Optimal Cutting Temperature (OCT) compound (Agar Scientific). Five-μm-thick sections were prepared and sectioned, followed by staining with hematoxylin plus eosin (H&E) and/or Oil Red O. Bright-field images of stained tissue sections were taken with the Keyence Microscope BZ-9000.

### Immunodetection of F4/80-postive cells in iWAT and eWAT

F4/80-postive cells in iWAT and eWAT of HFD adipo-Gi KO mice and control littermates were identified via immunostaining, using the anti-rat HRP-DAB Staining Kit (R&D System). Briefly, slides/sections were blocked with peroxide blocking reagent for 5 min, followed by a 15 min incubation with Serum-Blocking Reagent G. Subsequently, slides were incubated with Avidin Blocking Reagent for 15 min, rinsed with PBS, treated with Biotin Blocking Reagent for 15 min, and then incubated with the F4/80 primary antibody (1:200, Abcam) at 4 °C overnight. On the next day, slides were rinsed with PBS, treated with biotinylated secondary antibody for 60 min, and then incubated with High Sensitivity Streptavidin-HRP for 30 min. Finally, the slides were developed using DAB and counterstained with hematoxylin. Bright-field images of stained tissue sections were taken with a Keyence Microscope BZ-9000.

### RNA-seq study

Total RNA extracted from mature adipocytes (iWAT and eWAT) and BAT tissue of C57BL/6 mice maintained either on regular chow or high-fat diet for 12 weeks were used to construct high throughput sequencing libraries. RNAs with RIN > 8 (assessed by the Agilent 2100 Bioanalyzer system) were used to prepare transcriptome libraries using the NEBNext Ultra RNA library prep kit (New England Biolabs). High throughput RNA-sequencing was performed using a HiSeq 2500 instrument (Illumina) at the NIDDK Genomic Core Facility (NIH, Bethesda, MD). Raw reads were mapped to the mouse (mm9) genome. GPCRs were extracted from the RNA-seq data using R form. G_i_-coupled GPCRs were identified using the IUPHAR/BPS Guide to Pharmacology Database (https://www.guidetopharmacology.org/). The RNA-seq data are viewable under GEO accession numbers GSE131861 (RC data) and GSE134914 (HFD data), respectively.

### Statistics

All data are expressed as means ± s.e.m. for the indicated number of observations. Prior to performing specific statistical tests, we performed tests for normality and homogeneity of variance. Data were then tested for statistical significance by one- or two-way ANOVA, followed by the indicated post hoc tests, or by using a two-tailed unpaired Student’s *t* test, as appropriate. A *P* value of <0.05 was considered statistically significant. The specific statistical tests that were used are indicated in the figure legends.

### Reporting summary

Further information on research design is available in the Nature Research Reporting Summary linked to this article.

## Supplementary information

Supplementary Information

Reporting Summary

## Data Availability

All sequencing data have been deposited in the GEO database with accession numbers GSE131861 (RC data) and GSE134914 (HFD data), respectively. A Source Data File that contains original data for all figures is available for this article. Source data are provided with this paper.

## Source data

Source Data
