# Supplementary material for: Adipocyte Gi signaling is essential for maintaining whole-body glucose homeostasis and insulin sensitivity
Source: Nat Commun. 2020 Jun 12;11:2995. doi: 10.1038/s41467-020-16756-x (PMC7293267; doi:10.1038/s41467-020-16756-x)
Supplement: Supplementary file 1 — Supplementary Information [file 41467_2020_16756_MOESM1_ESM.pdf]

Supplemental Information

Adipocyte G_i signaling is essential for maintaining whole-body glucose homeostasis and insulin sensitivity

Lei Wang, Sai P. Pydi, Lu Zhu, Luiz F. Barella, Yinghong Cui, Oksana Gavrilova, Kendra K. Bence, Cecile Vernochet, Jürgen Wess

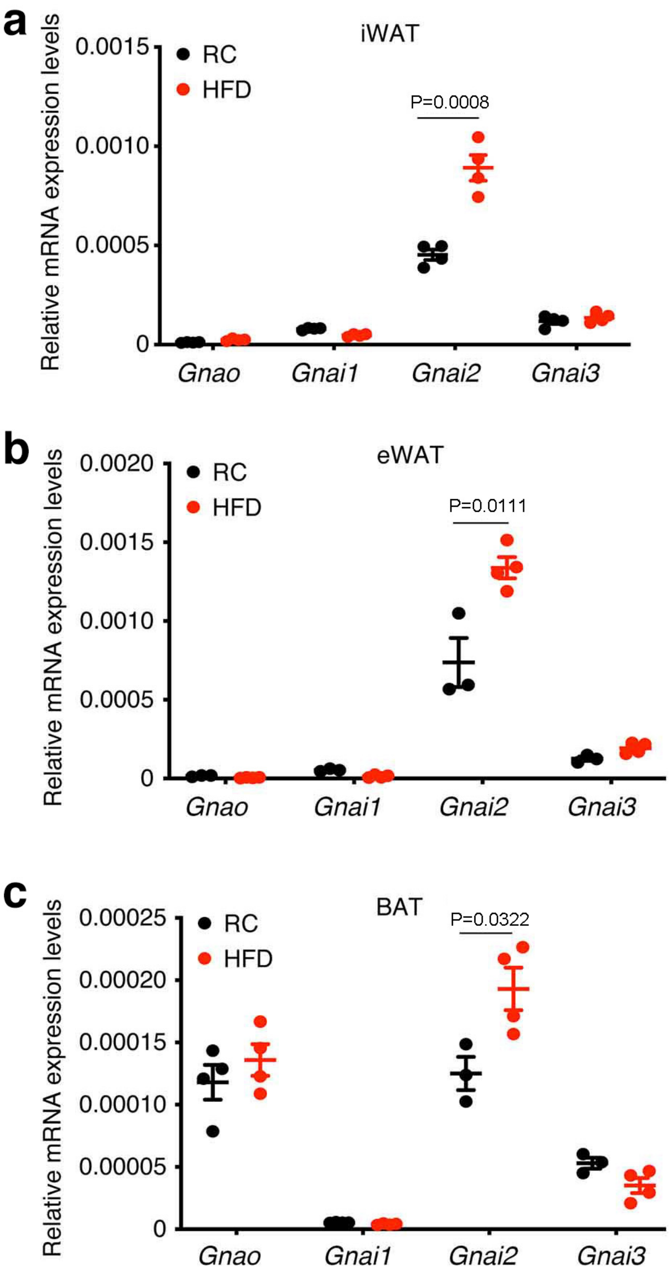

Supplementary Fig. 1 Expression of genes encoding G$\alpha_{i/o}$ proteins in different mouse fat depots. Wild-type C57BL-6NTac mice (males) were maintained on regular chow (RC) or a high-fat diet (HFD). RNA was isolated from inguinal white adipocytes (iWAT), epididymal white adipocytes (eWAT), and brown adipose tissue (BAT). **a-c,** Relative transcript levels of genes encoding G$\alpha_{i/o}$ proteins in iWAT (**a**), eWAT (**b**), and BAT (**c**). Relative gene expression was studied via qRT-PCR. Data are given as means ± s.e.m. (n=4 mice per group; two-tailed Mann-Whitney test). Source data are provided as a Source Data file.

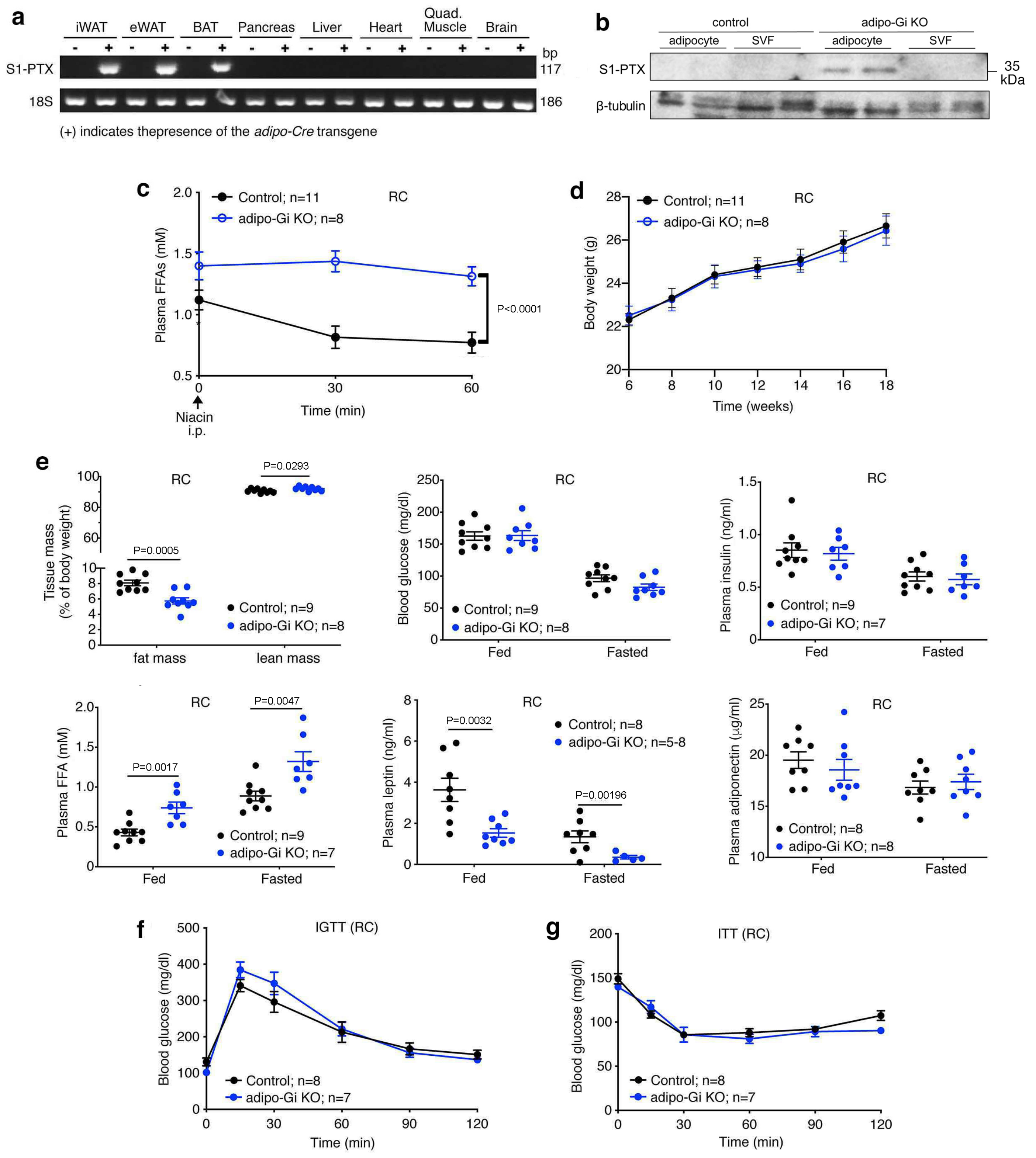

Supplementary Fig. 2. Metabolic analysis of adipo-Gi KO mice consuming regular chow (RC). a, RT-PCR analysis indicating that S1-PTX mRNA is selectively expressed in fat tissues of *adipoq-Cre ROSA26*^{PTX} mice (adipo-Gi KO mice). Three independent experiments gave similar results. **b,** Representative immunoblot showing S1-PTX protein (mass: 35 kDa) expression in adipocytes but not in the stromal vascular fraction (SVF) of adipose tissue (iWAT) isolated from adipo-Gi KO mice. Three independent experiments gave similar results. **c,** Plasma free fatty acid (FFA) levels of adipo-Gi KO and control mice after i.p. injection of niacin (100 mg/kg; mice had been fasted overnight). In control mice,

niacin lowers plasma FFA levels by activating G_i-coupled HCA_2 (GPR109A) receptors endogenously expressed by adipocytes. **d,** Body weight gain of mice maintained on RC. **e,** Body composition, fed and fasting blood glucose levels, and fed and fasting plasma insulin, FFA, leptin, and adiponectin concentrations of RC control and adipo-Gi KO mice. **f,** I.p. glucose tolerance test (IGTT, 2 g/kg glucose). **g,** Insulin tolerance test (ITT, 0.75 U/kg i.p.). Data are given as means ± s.e.m. (**c:** two-way ANOVA followed by Bonferroni's post-hoc test; **e:** two-tailed Student's t test). Source data are provided as a Source Data file.

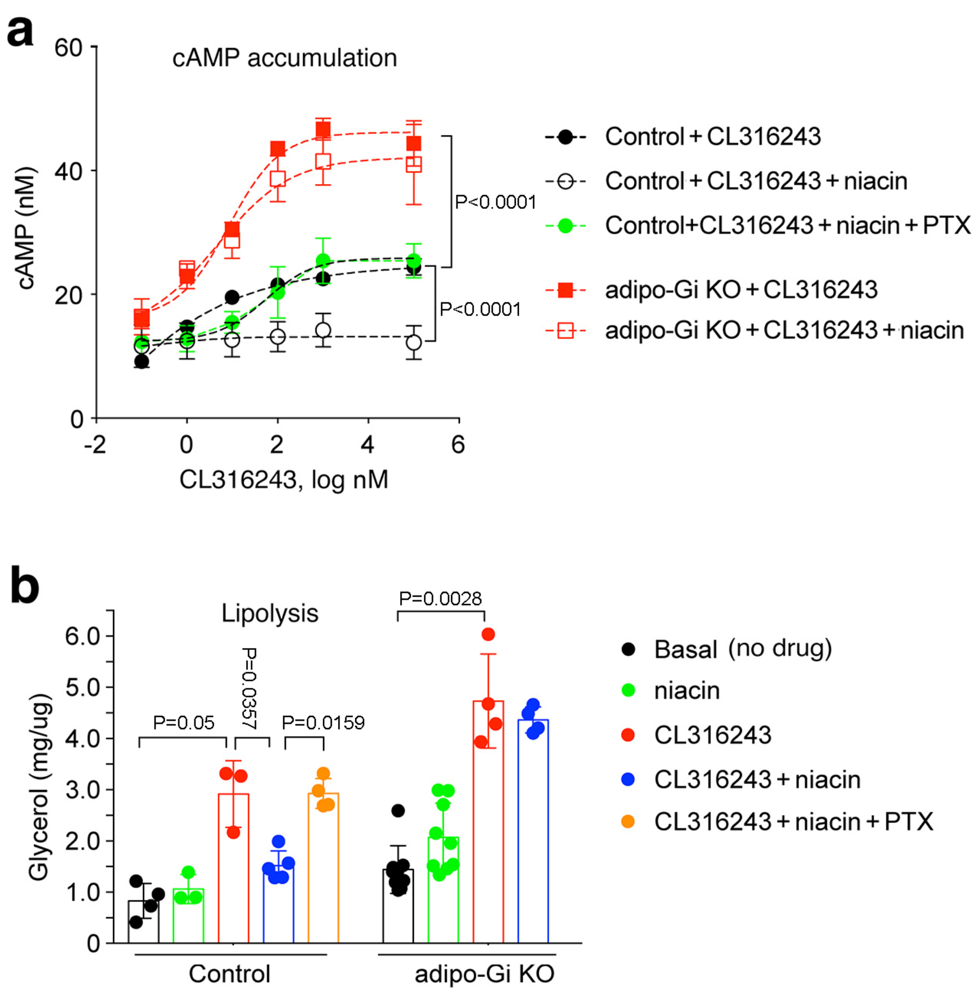

Supplementary Fig. 3. Adipocyte G_i signaling is inactivated in adipo-Gi KO mice. a, cAMP assay. Effect of niacin (10 µM) on CL316243 (1 nM)-induced stimulation of cAMP accumulation in primary adipocytes (iWAT) from control and adipo-Gi KO mice. While niacin acts as an agonist on G_i-coupled HCA_2 (GPR109A) receptors, CL316243 selectively activates G_s-coupled β3-adrenergic receptors. In control adipocytes, the inhibitory effect of niacin was completely blocked by PTX (100 ng/ml), confirming the involvement of G_i-type G proteins. In control adipocytes, the inhibitory effect of niacin was completely blocked by PTX (100 ng/ml), indicative of the involvement of G_i-type G proteins. In contrast, niacin-dependent inhibition of CL316243-induced stimulation of cAMP production was not observed with Gi KO adipocytes (n=6 per group). **b**, In vitro lipolysis assay. Primary adipocytes prepared from iWAT of control and adipo-Gi KO mice were left either untreated or incubated with 1 nM CL316243, 10 µM niacin, or 100 ng/ml PTX (control basal, n=4; control niacin, n=3; control CL316243, n=3; control CL316243+niacin, n=4; control CL316243+niacin+PTX, n=4; adipo-Gi KO basal, n=9; adipo-Gi KO niacin, n=9; adipo-Gi KO CL316243, n=4; adipo-Gi KO CL316243+niacin, n=4). The data obtained in this assay mirror the results of the cAMP assays (a). Data are presented as means ± s.e.m. (two-way ANOVA followed by Bonferroni's post-hoc test). Source data are provided as a Source Data file.

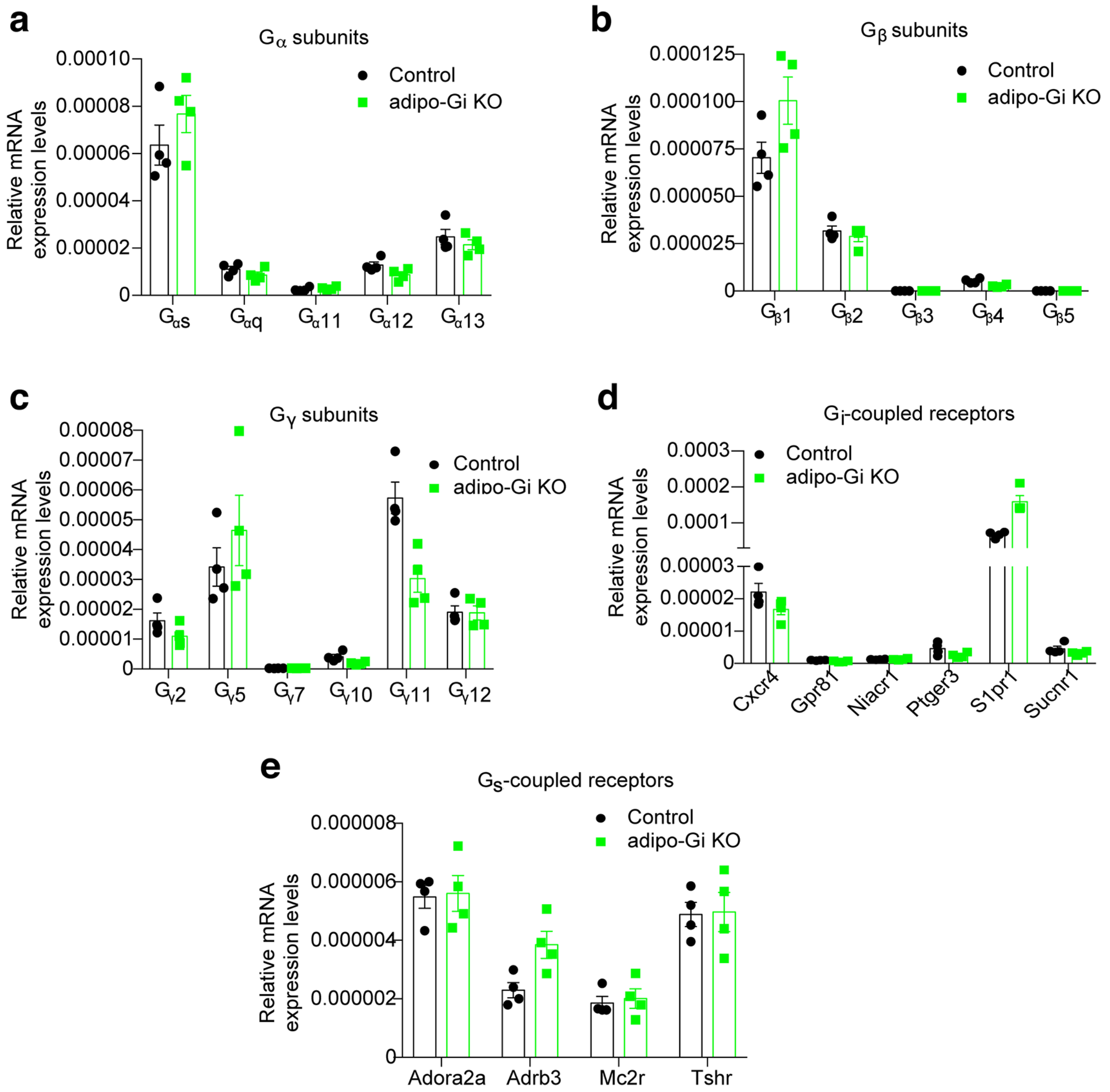

Supplementary Fig. 4. Loss of adipocyte G_i signaling does not affect the expression of other major adipocyte G protein subunits and G_i- and G_s-coupled receptors. a-e, Relative mRNA expression levels of G-protein α-subunits (**a**), G protein βγ-subunits (**b, c**), and major adipocyte G_i- and G_s-coupled receptors (**d, e**) in primary adipocytes (iWAT) prepared from control and adipo-Gi KO mice. Transcript levels were determined via qRT-PCR. mRNA expression data were normalized relative to the expression of β-actin (*Atcb*) or 18S rRNA using the ΔΔCt method. Data are presented as means ± s.e.m. (n=4 per group). Source data are provided as a Source Data file.

Cxcr4, chemokine (C-X-C motif) receptor 4, Gpr81, hydrocarboxylic acid receptor 1, Niacr, hydroxycarboxylic acid receptor 2, Ptger3, prostaglandin E receptor 3, S1pr1, sphingosine-1-phosphate receptor 1, Sucnr1, succinate receptor 1, Adora2a, adenosine A2a receptor; Adrb3, β3-adrenergic receptor; Mc2r, melanocortin 2 receptor; Tshr, thyroid stimulating hormone receptor.

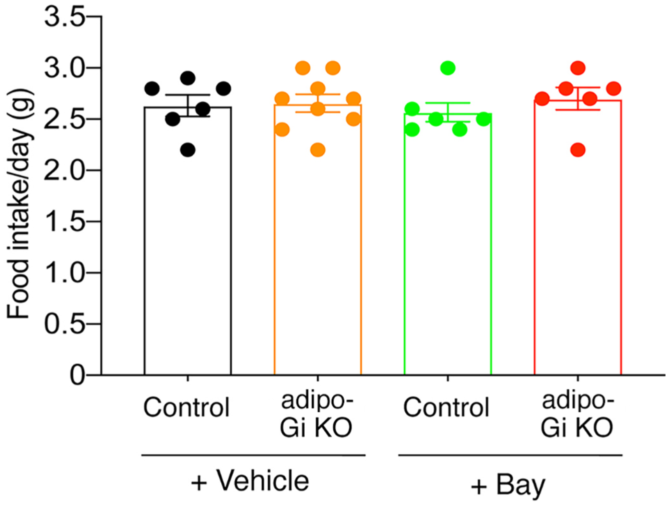

Supplementary Fig. 5. BAY 59-9435 (Bay) treatment of HFD control and adipo-Gi KO mice has no significant effect on food intake. Adipo-Gi KO mice and control littermates were housed individually and maintained on a HFD for 6 weeks. During the last two weeks of HFD feeding, mice received Bay (30 mg/kg) or vehicle (0.5% methylcellulose) via oral gavage once per day. Food intake was measured daily during the Bay/vehicle treatment period. Data are given as means ± s.e.m. (control+vehicle, n=6; adipo-Gi KO+vehicle, n=9; control+Bay, n=6; adipo-Gi KO+Bay, n=6). Note that Bay had no significant effect on food intake, as compared to vehicle-treated mice (two-way ANOVA followed by Bonferroni's post-hoc test). Source data are provided as a Source Data file.

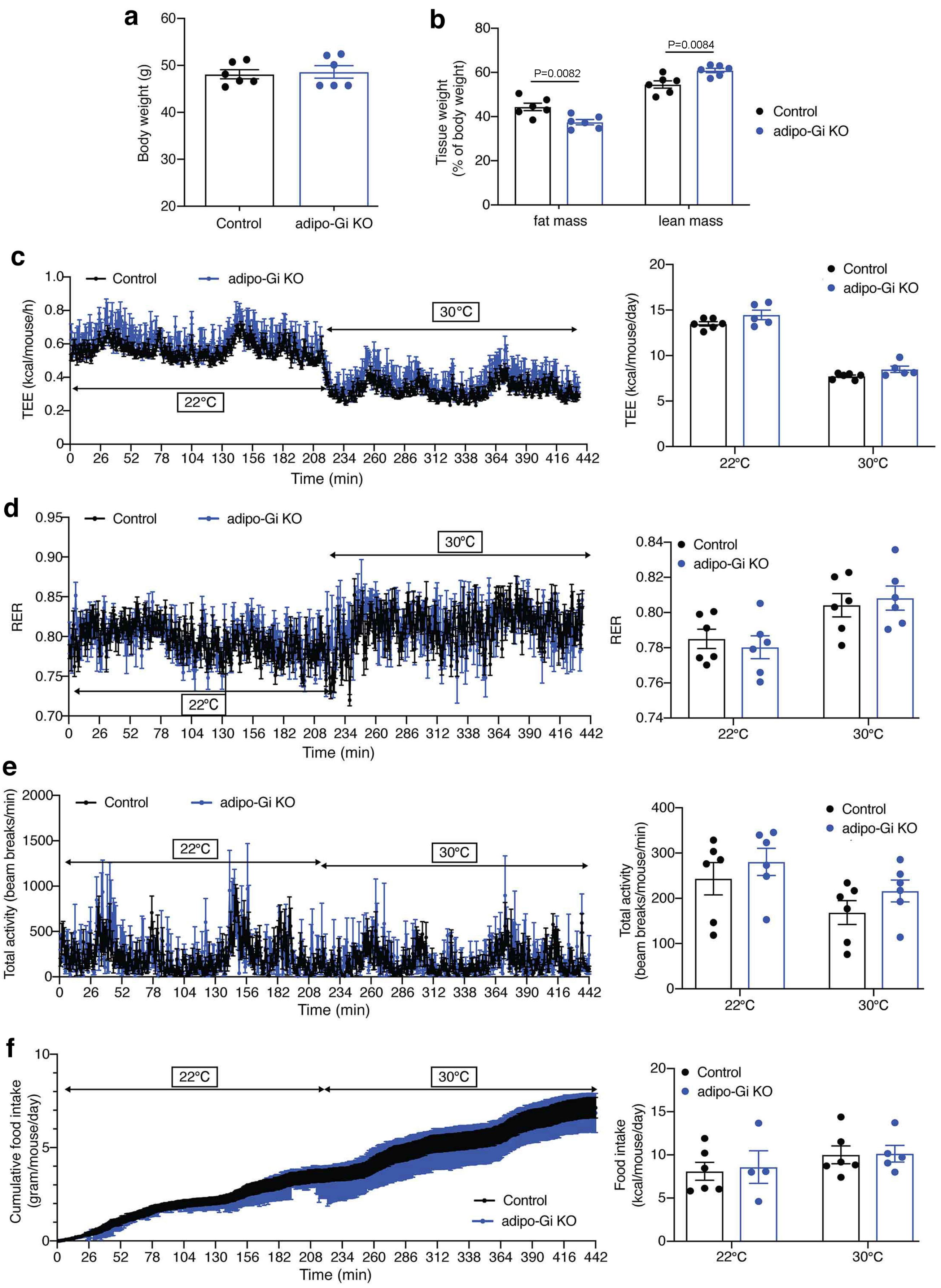

Supplementary Fig. 6. Lack of adipocyte G_i signaling has no effect on energy expenditure.

Control and adipo-Gi KO mice (males) that had been maintained on a HFD for 12 weeks were used to study energy expenditure via indirect calorimetry. **a,** Body weight after 12 weeks of HFD feeding. **b,** Analysis of body composition (fat vs. lean mass). **c,** Total energy expenditure (TEE) at room temperature (22 ºC) and at thermoneutrality (30 ºC). **d,** Respiratory exchange ratio (RER) at room temperature (22 ºC) and at thermoneutrality (30 ºC). **e,** Total locomotor activity at room temperature (22 ºC) and at thermoneutrality (30 ºC). **f,** Cumulative food intake at room temperature (22 ºC) and at thermoneutrality (30 ºC). Data represent means ± s.e.m. (n=6; two-tailed Student's t test). Source data are provided as a Source Data file.

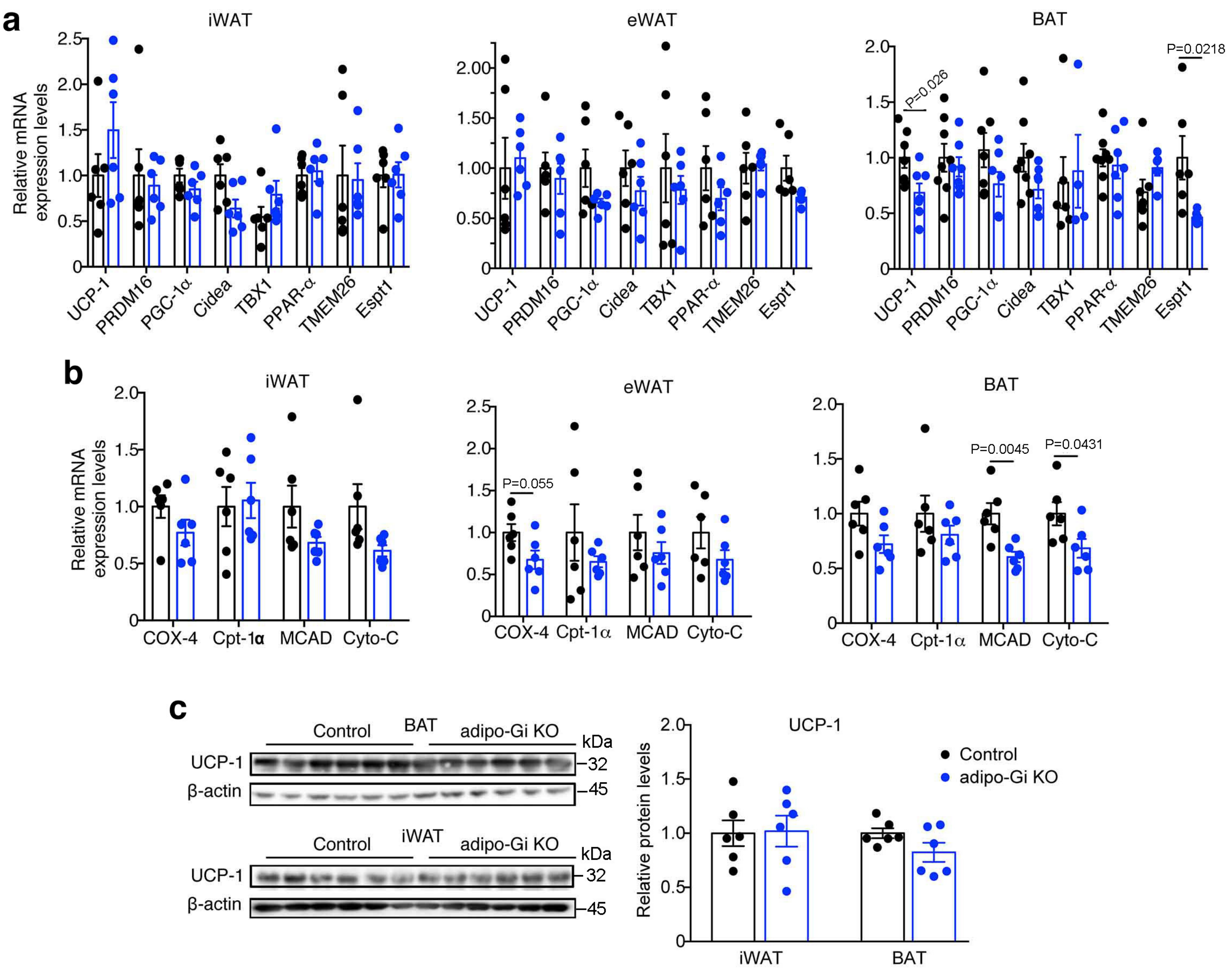

Supplementary Fig. 7. Lack of adipocyte G_i signaling has only minor effects on the expression of genes involved in adipose tissue browning/beiging and mitochondrial function. RNA prepared from different fat depots of HFD control and adipo-Gi KO mice (males) was subjected to qRT-PCR analysis using primers specific for the indicated genes. **a, b,** Expression levels of genes involved in the browning/beiging of adipose tissue (**a**) and of mitochondrial genes (**b**) in iWAT, eWAT and BAT. **c,** Immunoblots showing UCP-1 protein expression in iWAT and BAT from HFD control and adipo-Gi KO mice. Note that the lack of adipocyte G_i signaling has no effect on UCP-1 expression. Data are given as means ± s.e.m. (n=6; two-tailed Student's t test). Source data are provided as a Source Data file.

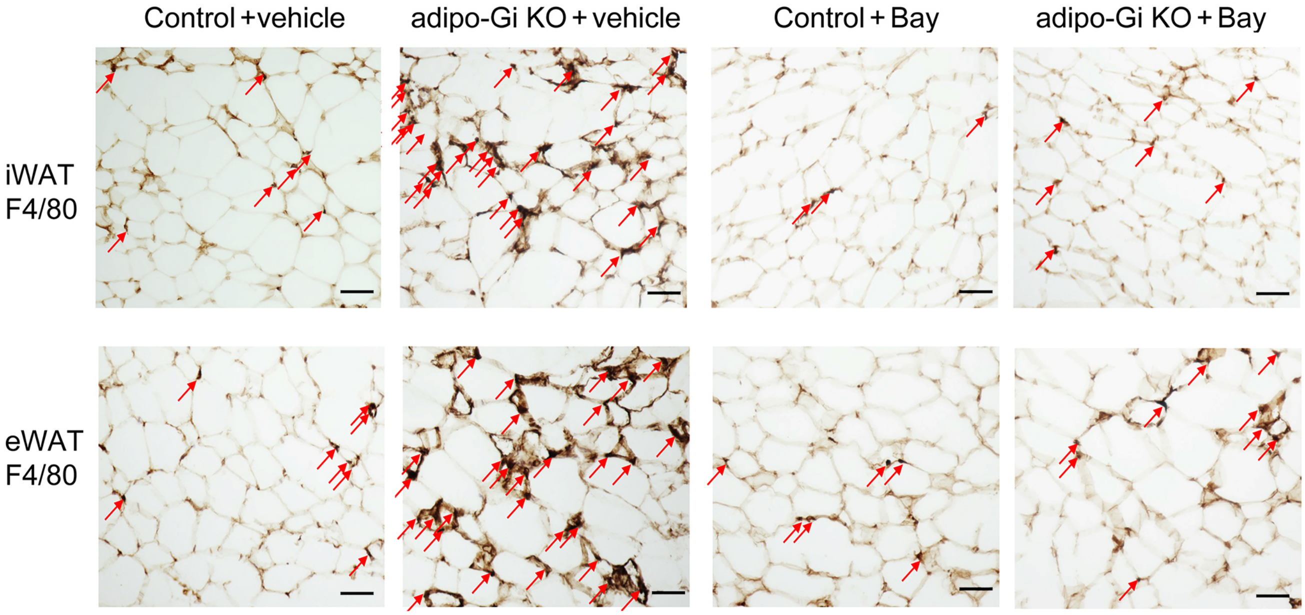

Supplementary Fig. 8. Identification of F4/80-postive cells in iWAT and eWAT sections from HFD adipo-Gi KO and control mice. Adipo-Gi KO mice and control littermates were maintained on a HFD for 6 weeks. During the last two weeks of HFD feeding, mice received Bay (30 mg/kg) or vehicle (0.5% methylcellulose) via oral gavage once per day. After the Bay/vehicle treatment period, iWAT and eWAT sections were prepared. Macrophages were identified by immunostaining using an anti-F4/80 antibody. Representative F4/80-positive cells are highlighted by red arrows. Note that Bay treatment dramatically reduced the number of F4/80-positive cells in Bay-treated HFD adipo-Gi KO mice (n=6 per group; Scale bars=50 μm). Source data are provided as a Source Data file.

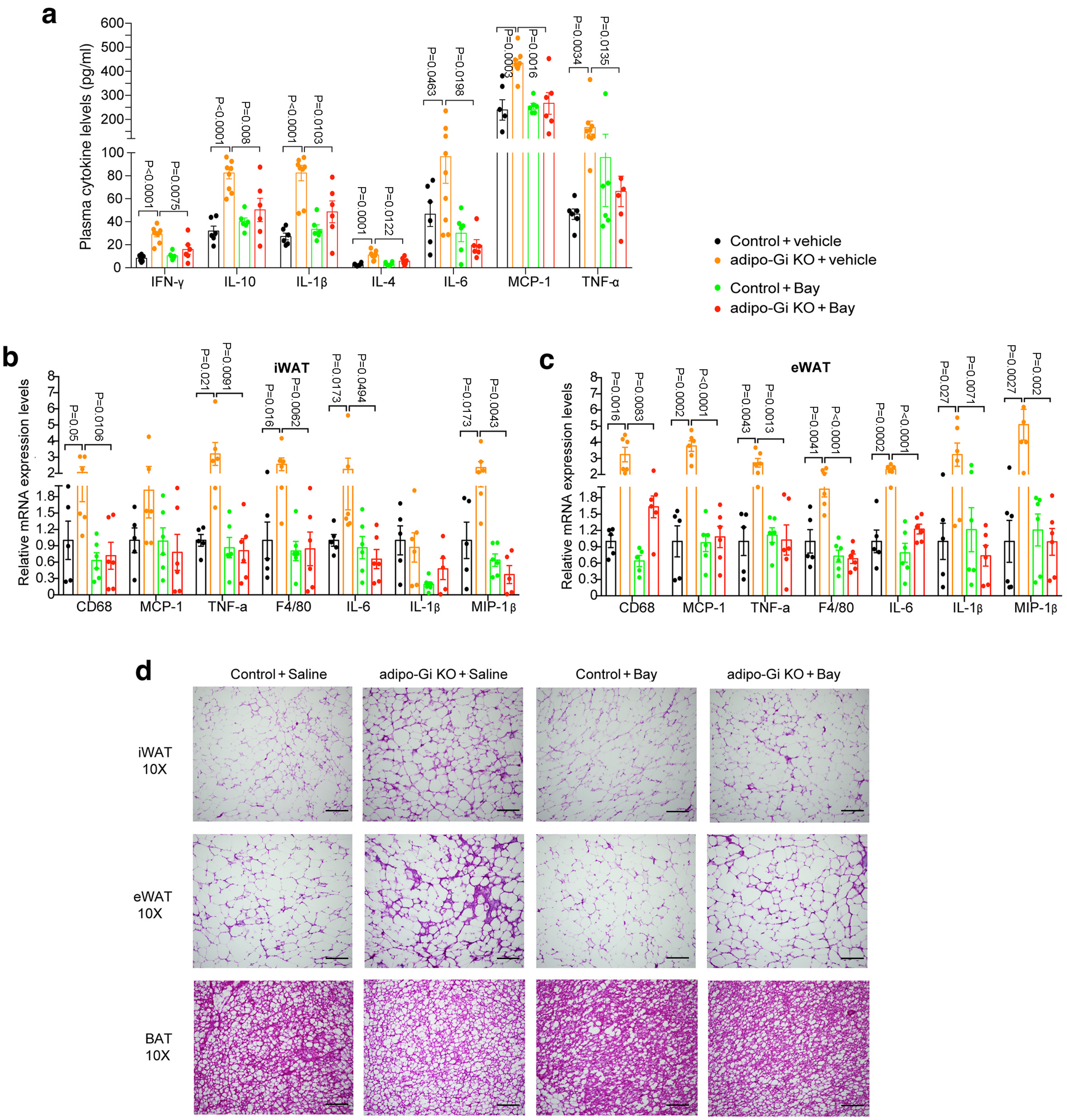

Supplementary Fig. 9. An HSL inhibitor reduces elevated levels of plasma cytokines and increased expression levels of proinflammatory genes displayed by HFD adipo-Gi KO mice. a, Plasma levels of inflammatory cytokines in adipo-Gi KO and control mice after 6 weeks of HFD feeding. During the last two weeks of HFD feeding, mice were treated daily with Bay (30 mg/kg/day via oral gavage) or vehicle (control+vehicle, n=6; adipo-Gi KO+vehicle, n=9; control+Bay, n=6; adipo-Gi KO+Bay, n=6). Interferon-γ, INF-γ; interleukin-10, IL-10; interleukin-1β, IL-1β; interleukin-6, IL-6; interleukin-4, IL-4; monocyte chemoattractant protein-1, MCP-1; tumor necrosis factor, TNF-α. **b, c,** Relative mRNA expression levels of inflammatory cytokines in iWAT (**b**) and eWAT (**c**) from adipo-Gi KO and control mice (n=6 per group) treated in the same way as described under (**a**). **d,** Representative sections of iWAT, eWAT, and BAT

stained with H&E (n=6 per group; scale bars=200 μm). Data are given as means ± s.e.m. (two-way ANOVA followed by Bonferroni's post-hoc test). Source data are provided as a Source Data file.

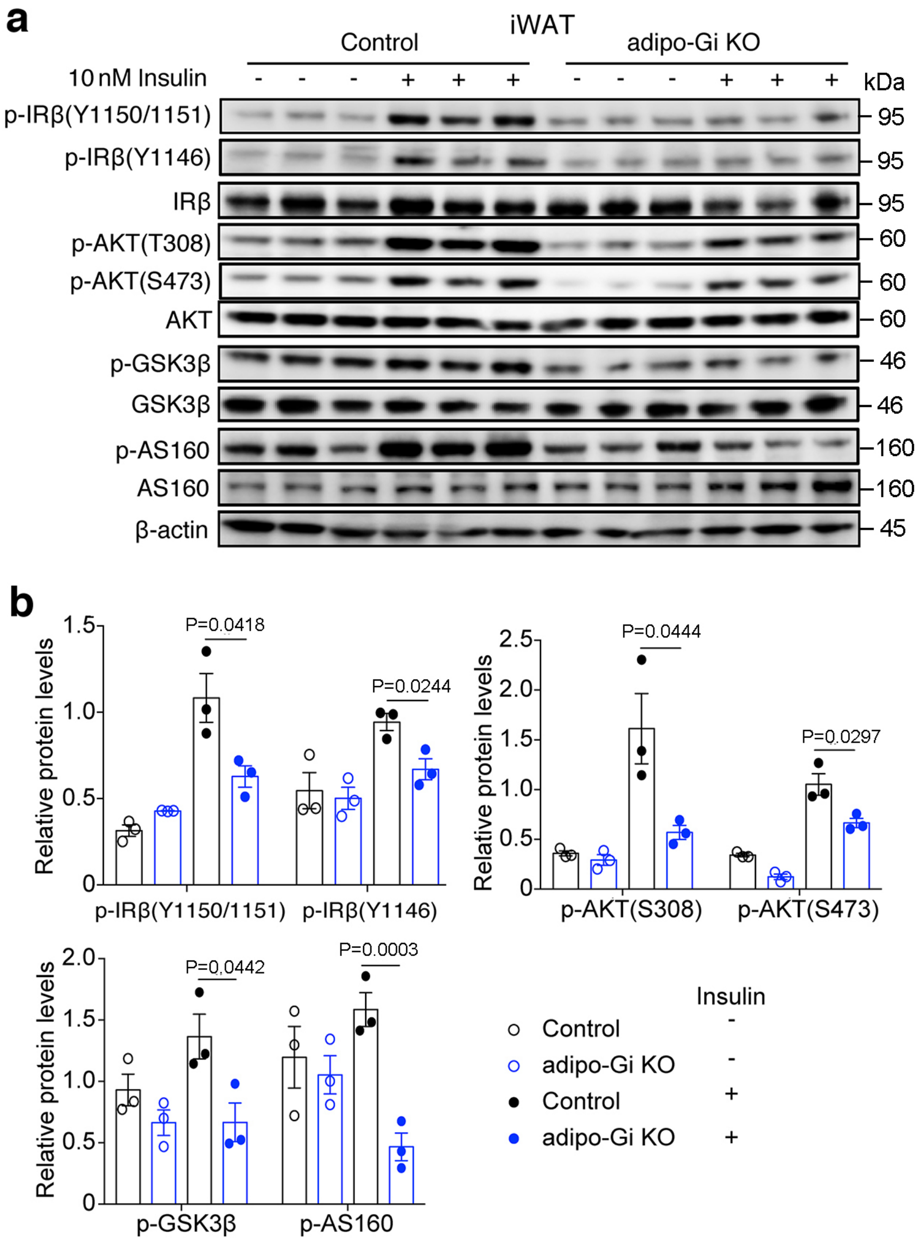

Supplementary Fig. 10. Immunoblotting studies indicating that insulin signaling is reduced in iWAT lacking functional G_i. HFD control and adipo-Gi KO mice were injected i.v. with either saline or insulin (5 U/mouse). iWAT was collected 5 min later and processed for immunoblotting studies. **a,** Western blots. **b,** Quantification of the Western blotting data. Data are given as means ± s.e.m. (n=3 per group; two-way ANOVA followed by Bonferroni's post-hoc test). Source data are provided as a Source Data file.

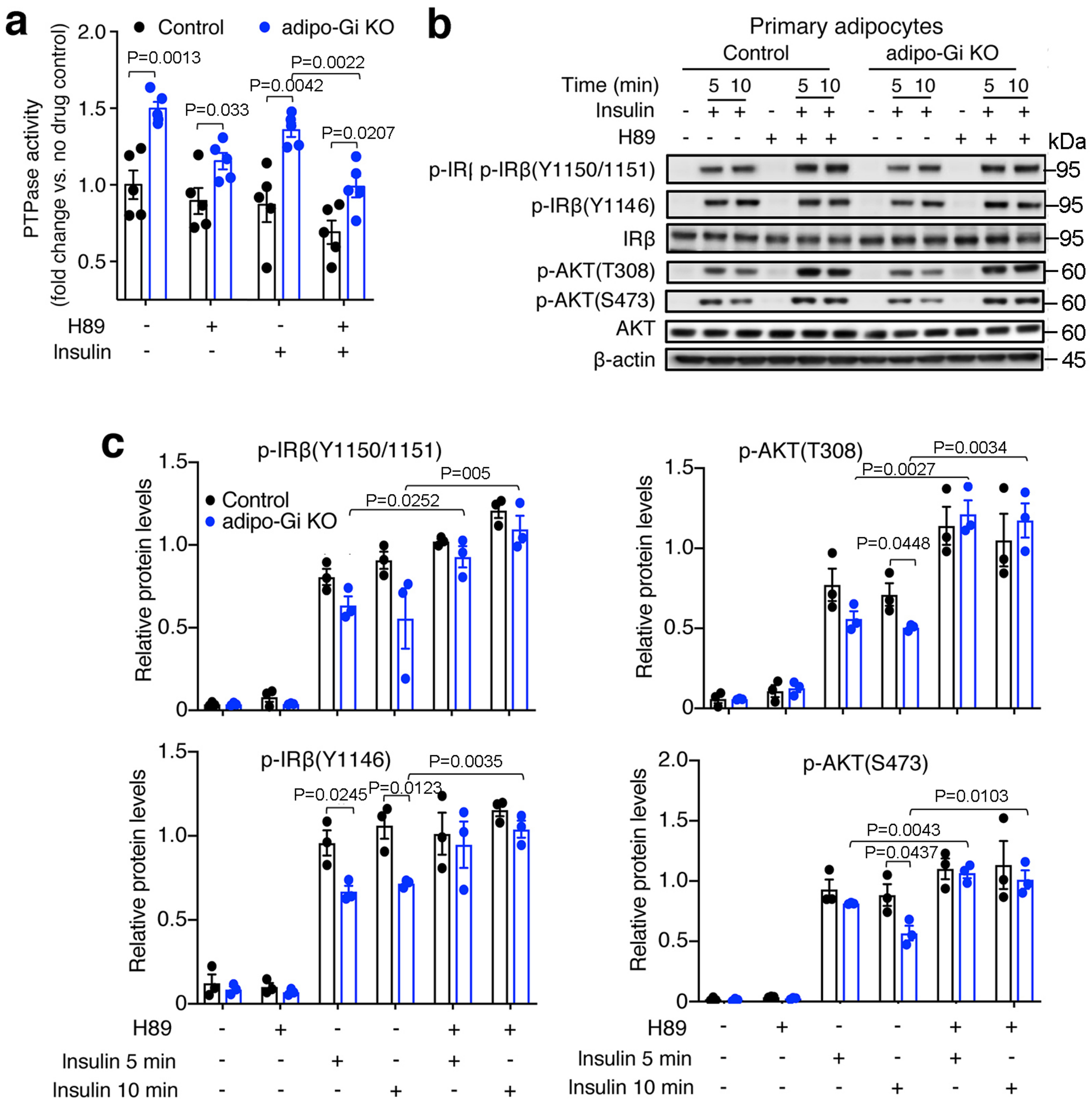

Supplementary Fig. 11. Effect of H89 on increased PTPase activity and impaired insulin signaling displayed by adipocytes lacking functional G_i. a, PTPase activity in primary adipocytes prepared from iWAT of adipo-Gi KO and control mice treated with either saline or insulin (10 nM) for 5 or 10 min, either in the absence or presence of PKA inhibitor (10 µM H89) (n=5 per group). **b,** Immunoblots showing the effect of H89 (10 µM) on insulin-stimulated phosphorylation of IRβ-Y1150/1151, IRβ-Y1146, AKT-T308, and AKT-S473 in primary adipocytes. Primary adipocytes from adipo-Gi KO and control mice were treated with 10 nM insulin for 5 or 10 min, either in the absence or presence of H89 (10 µM) (n=3). **c,** Quantification of Western blotting data. Data are presented as means ± s.e.m. (two-way ANOVA followed by Bonferroni's post-hoc test). Source data are provided as a Source Data file.

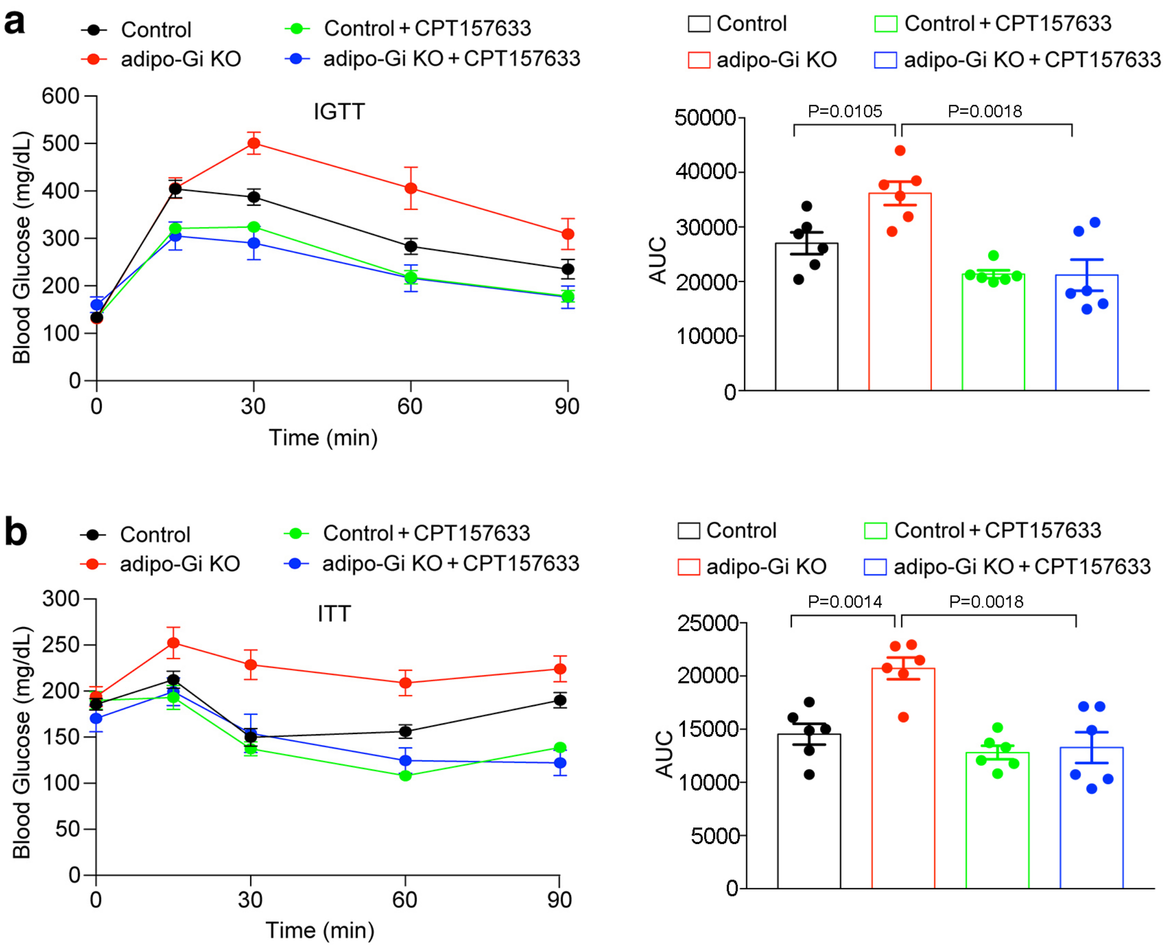

Supplementary Fig. 12. Treatment of HFD adipo-Gi KO mice with a PTP1B inhibitor restores proper glucose tolerance and insulin sensitivity. HFD control and adipo-Gi KO mice (age: 12 weeks, HFD during weeks 7-12) were fasted overnight for ~16 h. Mice were then injected with CPT157633 (5 mg/kg i.p.) or saline. Thirty min later, mice were subjected to glucose and insulin tolerance tests. **a,** Intraperitoneal glucose tolerance test (1 g/kg glucose, IGTT). **b,** Insulin tolerance test (1 U/kg, i.p.; ITT). AUC, area under curve. Data are given as means ± s.e.m. (n=6 mice per group; two-way ANOVA followed by Bonferroni's post-hoc test test). Source data are provided as a Source Data file.

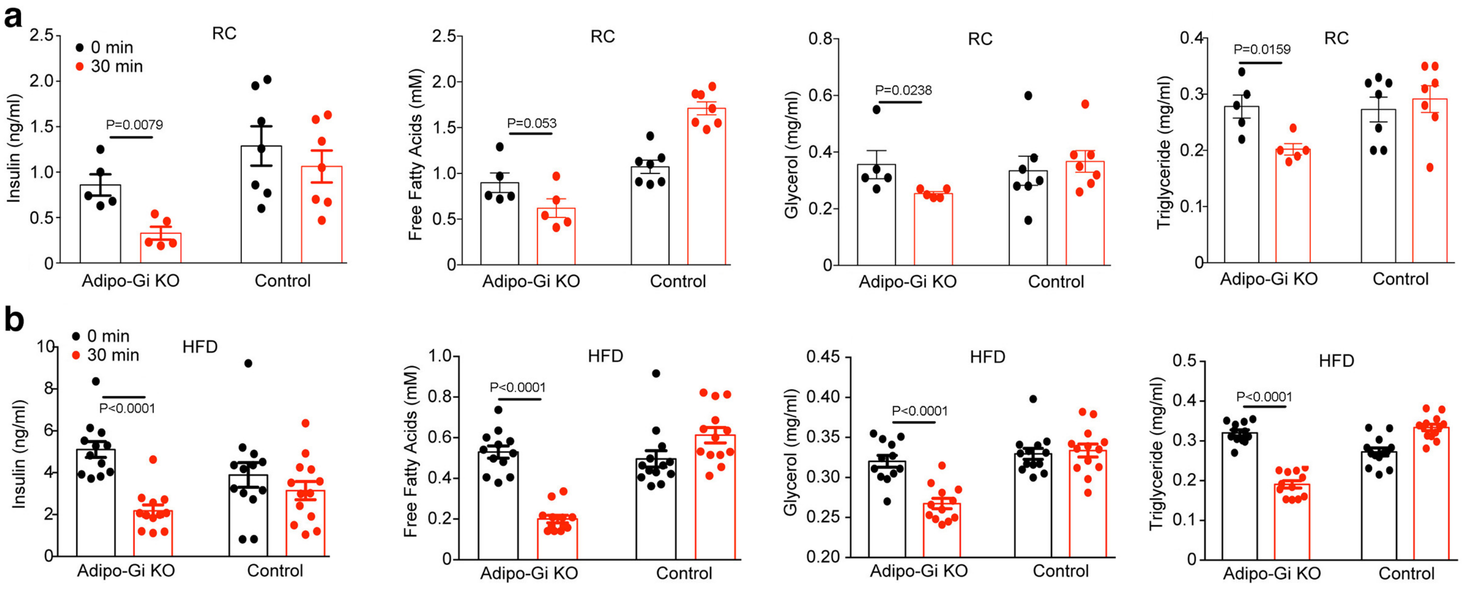

Supplementary Fig. 13. Activation of adipocyte G_i signaling causes reduced plasma insulin, FFA, glycerol, and triglyceride levels. Control and adipo-Gi KO mice (males) were maintained on regular chow (RC; age: 8-12 weeks) or a high-fat diet (HFD; age: 16 weeks) (mice were 6 weeks old when they were switched from RC to HFD). After a 4 hr fast, mice received an acute injection of CNO (10 mg/kg, i.p.). Plasma insulin, free fatty acid, glycerol, and triglyceride levels were determined immediately before and 30 min after CNO treatment. **a, b,** Effect of CNO on plasma insulin, free fatty acid, glycerol, and triglyceride levels in mice consuming RC (**a**) (control, n=7; adipo-GiD, n=5) or a HFD (**b**) (control, n=12; adipo-GiD, n=13). Data represent means ± s.e.m. (two-tailed Student's t test). Source data are provided as a Source Data file.

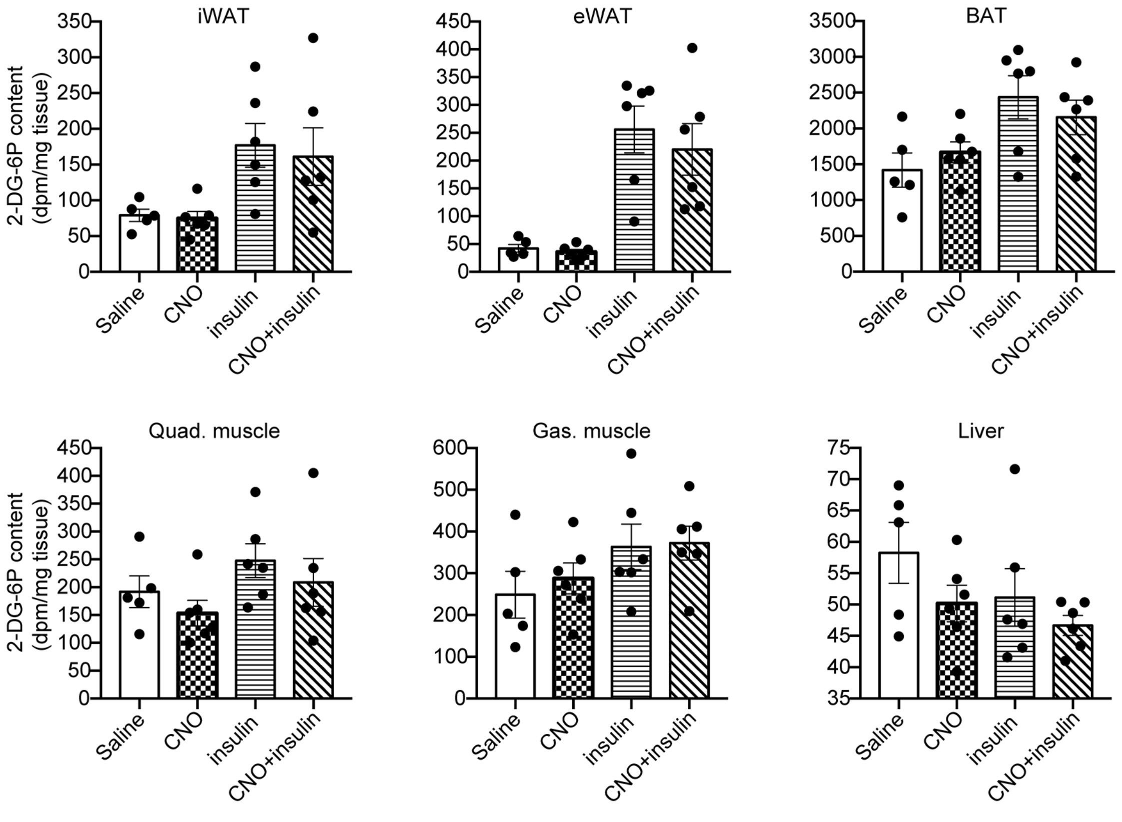

Supplementary Fig. 14. CNO has no effect on in vivo 2-deoxy-glucose (2-DG) uptake in control mice. In vivo 2-DG uptake was measured in iWAT, eWAT, BAT, quadriceps muscle (Quad.), gastrocnemius muscle (Gas.), and liver of control mice (8-week-old males) maintained on regular chow. Mice that had been fasted overnight were injected i.p with saline, 10 mg/kg CNO, 0.75 U/kg insulin, or 10 mg/kg CNO + 0.75 U/kg insulin (n=6 per group). Data are presented as means ± s.e.m. Insulin responses were not significantly affected by co-injection of CNO. Data were analyzed by two-way ANOVA followed by Bonferroni's post-hoc test. Source data are provided as a Source Data file.

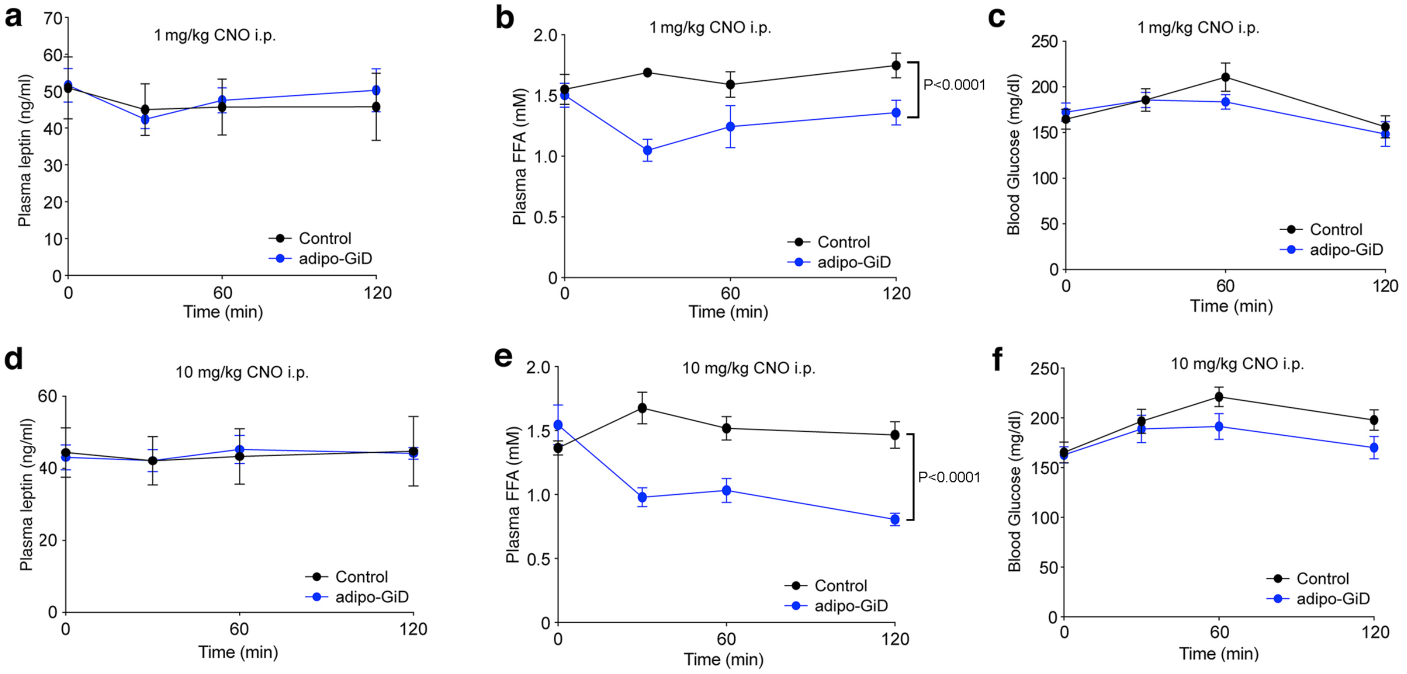

Supplementary Fig. 15. Effect of different CNO doses on plasma leptin, plasma free fatty acid (FFA), and blood glucose levels in adipo-GiD and control mice. Adipo-GiD mice and control littermates (32-week-old males) received a single i.p. injection of either 1 or 10 mg/kg of CNO, as indicated (diet: regular chow), followed by the measurement of plasma leptin, plasma FFA, and blood glucose over a 2 hr period. **a, d**, Plasma leptin levels. **b, e**, Plasma FFA levels. **c, f**, Blood glucose levels. Data are given as means ± s.e.m. (n=8 per group; two-way ANOVA followed by Bonferroni's post-hoc test). Source data are provided as a Source Data file.

Supplementary Table 1. RNA-Seq analysis of G_i-coupled GPCRs expressed in iWAT and eWAT (adipocytes) and BAT tissue

GPCR	Full name	Regular chow (RC, RPKM)	High fat diet (HFD, RPKM)	Fold change (RC vs. HFD)
Aplnr	apelin receptor	iWAT (2.51), eWAT (4.58), BAT (3.54)	iWAT (12.5), eWAT (3.99), BAT (5.43)	iWAT (4.98), eWAT (0.87), BAT (1.54)
Ccr1	chemokine (C-C motif) receptor 1	iWAT (1.05), eWAT (2.09), BAT (0.027)	iWAT (2.60), eWAT (10.50), BAT (0.052)	iWAT (2.456), eWAT (5.024), BAT (1.932)
Ccr10	chemokine (C-C motif) receptor 10	iWAT (0.728), eWAT (0.408), BAT (2.360)	iWAT (0.353), eWAT (0.104), BAT (2.86)	iWAT (0.485), eWAT (0.256), BAT (1.21)
Ccr2	chemokine (C-C motif) receptor 2	iWAT (0.382), eWAT (1.584), BAT (0.618)	iWAT (1.95), eWAT (4.76), BAT (0.658)	iWAT (5.11), eWAT (3.00), BAT (1.07)
Ccr3	chemokine (C-C motif) receptor 3	iWAT (0.027), eWAT (0.056), BAT (0.002)	iWAT (0.419), eWAT (2.41), BAT (0.001)	iWAT (15.5), eWAT (42.7), BAT (2.58)
Ccr4	chemokine (C-C motif) receptor 4	iWAT (0.006), eWAT (0.002), BAT (0.007)	iWAT (0.010), eWAT (0.002), BAT (0.018)	iWAT (1.52), eWAT (0.720), BAT (2.45)
Ccr5	chemokine (C-C motif) receptor 5	iWAT (0.240), eWAT (0.699), BAT (0.189)	iWAT (2.68), eWAT (9.76), BAT (0.245)	iWAT (11.2), eWAT (13.5), BAT (1.30)
Ccr6	chemokine (C-C motif) receptor 6	iWAT (0.006), eWAT (0.005), BAT (0.010)	iWAT (0.013), eWAT (0.002), BAT (0.010)	iWAT (2.25), eWAT (0.422), BAT (0.973)
Ccr7	chemokine (C-C motif) receptor 7	iWAT (0.315), eWAT (0.457), BAT (0.093)	iWAT (0.860), eWAT (0.457), BAT (0.164)	iWAT (2.73), eWAT (0.457), BAT (1.75)
Ccr9	chemokine (C-C motif) receptor 9	iWAT (0.261), eWAT (0.329), BAT (0.280)	iWAT (0.347), eWAT (0.361), BAT (0.243)	iWAT (1.33), eWAT (1.10), BAT (0.870)
Ccrl2	chemokine (C-C motif) receptor-like 2	iWAT (1.78), eWAT (8.19), BAT (0.453)	iWAT (18.4), eWAT (65.7), BAT (0.804)	iWAT (10.3), eWAT (8.02), BAT (1.77)
Chrm2	cholinergic receptor, muscarinic 2	iWAT (0.003), eWAT (0.027), BAT (0.003)	iWAT (0.007), eWAT (0.003), BAT (0.002)	iWAT (2.38), eWAT (0.118), BAT (0.617)
Chrm4	cholinergic receptor, muscarinic 4	iWAT (0.155), eWAT (0.108), BAT (0.005)	iWAT (1.589), eWAT (0.122), BAT (0.015)	iWAT (10.2), eWAT (1.12), BAT (2.69)
Cmklr1	chemokine-like receptor 1	iWAT (43.2), eWAT (33.9), BAT (1.40)	iWAT (31.0), eWAT (21.2), BAT (1.02)	iWAT (0.72), eWAT (0.62), BAT (0.727)
Cnr1	cannabinoid receptor 1	iWAT (4.17), eWAT (2.16), BAT (0.084)	iWAT (1.57), eWAT (0.736), BAT (0.072)	iWAT (0.378), eWAT (0.341), BAT (0.864)
Cx3cr1	chemokine (C-X3-C motif) receptor 1	iWAT (0.055), eWAT (0.180), BAT (0.335)	iWAT (0.219), eWAT (0.709), BAT (0.371)	iWAT (3.95), eWAT (3.93), BAT (1.11)
Cxcr1	chemokine (C-X-C motif) receptor 1	iWAT (0.006), eWAT (0.006), BAT (0.024)	iWAT (0.020), eWAT (--), BAT (0.064)	iWAT (3.19), eWAT (--), BAT (2.68)
Cxcr2	chemokine (C-X-C motif) receptor 2	iWAT (0.012), eWAT (0.019), BAT (0.016)	iWAT (0.029), eWAT (0.033), BAT (0.026)	iWAT (2.40), eWAT (1.74), BAT (1.61)
Cxcr3	chemokine (C-X-C motif) receptor 3	iWAT (0.100), eWAT (0.061), BAT (0.138)	iWAT (0.119), eWAT (0.616), BAT (0.052)	iWAT (1.19), eWAT (10.1), BAT (0.373)
Cxcr4	chemokine (C-X-C motif) receptor 4	iWAT (2.23), eWAT (7.00), BAT (0.571)	iWAT (26.3), eWAT (82.3), BAT (1.07)	iWAT (11.8), eWAT (7.00), BAT (1.88)
Cxcr5	chemokine (C-X-C motif) receptor 5	iWAT (0.183), eWAT (0.183), BAT (0.102)	iWAT (0.260), eWAT (0.095), BAT (0.133)	iWAT (1.42), eWAT (0.520), BAT (0.132)
Cxcr6	chemokine (C-X-C motif) receptor 6	iWAT (0.021), eWAT (0.030), BAT (0.127)	iWAT (0.058), eWAT (0.063), BAT (0.063)	iWAT (2.73), eWAT (2.10), BAT (0.491)
Cxcr7	atypical chemokine receptor 3	iWAT (4.13), eWAT (10.9), BAT (18.1)	iWAT (14.4), eWAT (8.55), BAT (24.3)	iWAT (3.48), eWAT (0.784), BAT (1.34)
Gabbr1	gamma-aminobutyric acid (GABA) B receptor, 1	iWAT (0.722), eWAT (0.967), BAT (1.12)	iWAT (0.849), eWAT (0.333), BAT (1.53)	iWAT (1.18), eWAT (0.344), BAT (1.368)
Gpr183	G protein-coupled receptor 183	iWAT (0.150), eWAT (0.364), BAT (0.139)	iWAT (2.44), eWAT (9.43), BAT (0.169)	iWAT (16.2), eWAT (25.9), BAT (1.21)
Gpr20	G protein-coupled receptor 20	iWAT (0.008), eWAT (0.003), BAT (0.065)	iWAT (0.026), eWAT (0.022), BAT (0.078)	iWAT (3.17), eWAT (6.76), BAT (1.19)
Gpr30	G protein-coupled estrogen receptor 1	iWAT (0.268), eWAT (0.311), BAT (0.462)	iWAT (1.32), eWAT (0.235), BAT (0.796)	iWAT (4.94), eWAT (0.756), BAT (1.72)
Gpr34	G protein-coupled receptor 34	iWAT (0.290), eWAT (0.753), BAT (0.286)	iWAT (2.81), eWAT (7.98), BAT (0.287)	iWAT (9.69), eWAT (10.6), BAT (1.00)
Gpr44	prostaglandin D2 receptor 2	iWAT (0.276), eWAT (0.314), BAT (0.137)	iWAT (0.488), eWAT (0.654), BAT (0.228)	iWAT (1.77), eWAT (2.08), BAT (1.05)
Gpr81	hydrocarboxylic acid receptor 1	iWAT (37.8), eWAT (32.6), BAT (13.5)	iWAT (15.6), eWAT (8.36), BAT (1.13)	iWAT (0.413), eWAT (0.256), BAT (0.084)
Gpr84	G protein-coupled receptor 84	iWAT (0.064), eWAT (0.215), BAT (0.003)	iWAT (0.641), eWAT (2.38), BAT (0.007)	iWAT (9.94), eWAT (11.1), BAT (1.88)
Ltb4r2	leukotriene B4 receptor 2	iWAT (0.014), eWAT (0.068), BAT (0.006)	iWAT (0.028), eWAT (0.022), BAT (0.008)	iWAT (2.08), eWAT (0.327), BAT (1.45)
Niacr1	hydroxycarboxylic acid receptor 2	iWAT (61.7), eWAT (18.5), BAT (2.91)	iWAT (19.9), eWAT (7.01), BAT (0.417)	iWAT (0.322), eWAT (0.380), BAT (2.915)
Npy1r	neuropeptide Y receptor Y1	iWAT (0.117), eWAT (0.125), BAT (0.251)	iWAT (0.419), eWAT (0.193), BAT (0.262)	iWAT (3.575), eWAT (1.545), BAT (1.045)

P2ry12	purinergic receptor P2Y, G-protein coupled 12	iWAT (0.261), eWAT (0.260), BAT (0.156)	iWAT (0.385), eWAT (1.22), BAT (0.185)	iWAT (1.48), eWAT (4.68), BAT (1.18)
P2ry14	purinergic receptor P2Y, G-protein coupled, 14	iWAT (0.071), eWAT (0.109), BAT (0.109)	iWAT (0.349), eWAT (0.900), BAT (0.109)	iWAT (4.90), eWAT (8.26), BAT (0.999)
Ptger3	prostaglandin E receptor 3	iWAT (19.9), eWAT (20.5), BAT (15.2)	iWAT (33.6), eWAT (10.2), BAT (14.8)	iWAT (1.69), eWAT (0.499), BAT (0.973)
S1pr1	sphingosine-1-phosphate receptor 1	iWAT (14.8), eWAT (28.0), BAT (17.928)	iWAT (57.2), eWAT (22.8), BAT (27.9)	iWAT (3.86), eWAT (0.814), BAT (1.56)
Sstr2	somatostatin receptor 2	iWAT (0.010), eWAT (0.049), BAT (0.008)	iWAT (0.395), eWAT (1.77), BAT (0.012)	iWAT (41.6), eWAT (36.3), BAT (1.59)
Sstr4	somatostatin receptor 4	iWAT (0.154), eWAT (0.189), BAT (0.156)	iWAT (0.095), eWAT (0.069), BAT (0.299)	iWAT (0.617), eWAT (0.365), BAT (1.919)
Sucnr1	succinate receptor 1	iWAT (37.0), eWAT (57.3), BAT (2.23)	iWAT (31.7), eWAT (14.2), BAT (4.10)	iWAT (0.856), eWAT (0.247), BAT (1.84)

RPKM, Reads Per Kilobase of transcript, per Million mapped reads. RPKM data are given as mean values of 6 samples. Adipocytes/fat tissue were isolated from 16-week-old wild-type male C57BL/6NTac mice raised on regular chow (RC) or maintained on a high-fat diet (HFD) for 12 weeks (n=6 per group).

Supplementary Table 2. Reagents and animals used in this study

Reagent	Source	Catalog # (identifier)
**Antibodies**		
Phospho-Insulin Receptor beta (Tyr1150/1151)	Cell Signaling	3024
Phospho-Insulin Receptor beta (Tyr1146)	Cell Signaling	3021
Insulin Receptor beta	Cell Signaling	3020
Phospho-IRS1 (Ser612)	Cell Signaling	3203
IRS1	Cell Signaling	2382
PI3 Kinase p85	Cell Signaling	4257
Phospho-PI3 Kinase p85 (Tyr458)/p55 (Tyr199)	Cell Signaling	4228
Phospho-Akt (Thr308)	Cell Signaling	2965
Phospho-Akt (Ser473)	Cell Signaling	4060
Akt	Cell Signaling	9272
Phospho-FoxO1 (Ser256)	Cell Signaling	9461
FoxO1	Cell Signaling	2880 (clone C29H4)
Phospho-GSK-3β (Ser9)	Cell Signaling	9336
GSK-3α/β (D75D3)	Cell Signaling	5676
Phospho-HSL (Ser563)	Cell Signaling	4139
Phospho-HSL (Ser660)	Cell Signaling	4126
HSL	Cell Signaling	4107
Phospho-ATGL (Ser406)	Abcam	ab135093
ATGL	Abcam	2138
Phospho-AS160 (Thr642)	Millipore Sigma	ABS271
AS160	Millipore Sigma	07-741
Phospho-NF-κB p65	Cell Signaling	3033
NF-κB p65	Cell Signaling	8242
S1-PTX	EpiGentek	A53718
β–Tubulin	Cell Signaling	86298
F4/80	Abcam	Ab6640
UCP-1	Abcam	Ab10983
β-Actin	Cell Signaling	4970
**Chemicals**		
Clozapine N-oxide (CNO)	Toronto Research Chemicals	C587520
BAY 59-9435	Pfizer	Gift by Pfizer Inc.
CPT157633	Cold Spring Harbor	Provided by Nicholas K. Tonks
D-Glucose	Macron Fine Chemical	4912-12
Insulin (human)	Sigma-Aldrich	I9278
Pyruvate (sodium salt)	Sigma-Aldrich	P2256
Dexamethasone	Sigma-Aldrich	D4902
Phenylmethylsulfonyl fluoride	ThermoFisher Scientific	36978
2-Deoxy-D-glucose	Sigma-Aldrich	D8375
3-Isobutyl-1-methylxanthine (IBMX)	Sigma-Aldrich	I5879
Troglitazone	Sigma-Aldrich	T2573
Indomethacin	Sigma-Aldrich	I7378
Cytochalasin B	Sigma-Aldrich	C6762
CL-316243	Sigma-Aldrich	C5976
^{3}H-2-deoxy-glucose	PerkinElmer	NET328A001MC
^{14}C-2-deoxy-glucose	PerkinElmer	NEC720A050U
Bovine serum albumin (fatty acid-free)	Sigma-Aldrich	A7030
Collagen I	Sigma-Aldrich	C0130
Triton X-100	ThermoFisher Scientific	BP151
Tween 20	ThermoFisher Scientific	BP337
SuperSignal™ West Pico Chemiluminescent Substrate	ThermoFisher Scientific	34080

TRIzol	Invitrogen	15596026
Isoproterenol	Sigma-Aldrich	35100 USP
Pertussis toxin	Sigma-Aldrich	P7208
Niacin (nicotinic acid)	Sigma-Aldrich	PHR1276
Power SYBR Green PCR Master Mix	Applied Biosystems	4367659
TaqMan Universal PCR Master Mix	Applied Biosystems	4304437
NuPAGE LDS sample buffer	ThermoFisher Scientific	NP0007
Nitrocellulose membranes	BioRad	1704158
NuPAGE 4-12% Bis-Tris protein gel	Invitrogen	NP0336BOX
NuPAGE MOPS SDS Running Buffer (20 x)	ThermoFisher Scientific	NP0001
Oil Red O	Sigma-Aldrich	O0625
**Assay kits**		
BCA protein assay kit	Pierce	23225
RNase-free DNase I	Qiagen	79254
RNase mini kit	Qiagen	74104
SuperScript III-First-Strand Synthesis System for RT-PCR	Invitrogen	18080400
cOmplete EDTA-free protease inhibitor cocktail	Sigma-Aldrich	11873580001
Ultra-sensitive mouse insulin ELISA kit	Crystal Chem	90080
cAMP ELISA kit	Cayman Chemicals	581001
Mouse/Rat Leptin Quantikine ELISA Kit	R&D Systems	MOB00
Mouse Adiponectin/Acrp30 Quantikine ELISA kit	R&D Systems	MRP300
cAMP Dynamic kit	Cisbio Bioassays	62AM4PE
Free Glycerol reagent	Sigma-Aldrich	F6428
HR Series NEFA-HR(2)	FUJIFILM Wako Diagnostics	999-34691, 995-34791 991-34891, 993-35191 276-76491
Triglyceride Reagent	Sigma-Aldrich	T2449
Bio-Rad Protein Assay kit	BioRad	5000001
PTP Activity Assay kit (Fluorometric)	BioVision	K829-100
Bio-Plex Multiplex Immunoassay System	Bio-Rad	custom made
Anti-Rat HRP-DAB Staining kit	R&D Systems	CTS017
**Experimental Models: Organisms/Strains**		
Adipoq-Cre mice	The Jackson Laboratory (Eguchi et al., 2011)	Stock# 010803
ROSA26-PTX$^{flox/flox}$ mice	Provided by Dr. Shaun Coughlin (Regard et al., 2007)	NA
Rosa26-LSL-hM4Di mice	Provided by Dr. Bryan Roth (Zhu et al., 2016)	NA
**Oligonucleotides**<br>A complete list is provided in Table S2		
**Software and Algorithms**		
Prism 7	Graph Pad	https://www.graphpad.com/scientific-software/prism/
ImageJ	NIH	http://imagej.net/Adiposoft
Other		

Supplementary Table 3. Primers and probes used for quantitative real-time PCR studies

Gene Target	Species	Primer sequence
GiD	mouse	Probe: 5'-/56-FAM/CCTGGTCAC/ZEN/GTCATCATCCCACAA/3IABkFQ/-3'<br>Primer 1: 5'-TACGCTATGGCCAACTTCAC-3'<br>Primer 2: 5'-GACCATTTCCACCGTCTCATAG-3' (Taqman primers and probe)
β-actin	mouse	Probe: 5'-/56-FAM/TGGCATTGT/ZEN/TACCAACTGGGACGA/3IABkFQ/-3'<br>Primer 1: 5'-GAGGTATCCTGACCCTGAAGTA-3'<br>Primer 2: 5'-CACACGCAGCTCATTGTAGA-3' (Taqman primers and probe)
β-actin	mouse	Forward: 5'-GATATCGCTGCGCTGGTCGTC-3'<br>Reverse: 5'-ACGCAGCTCATTGTAGAAGGTGTGG-3'
Ptxs1<br>(S1-PTX)	Bordetella pertussis	Forward: 5'-GGTCTATCTCGAACATCGCA-3'<br>Reverse: 5'-GCGCCGTAGAAATTGTTGT-3'
18S rRNA	mouse	Forward: 5'-CGGCTACCACATCCAAGGAA-3'<br>Reverse: 5'-GCTGGAATTACCGCGGCT-3'
Cd68	mouse	Forward: 5'-GGAAGAAAGGCTTGGGGCAT-3'<br>Reverse: 5'-ATTCCACCGCCATGTAGTCC-3'
Mcp1<br>(MCP-1)	mouse	Forward: 5'-AGCTGTAGTTTTTGTCACCAAGC-3'<br>Reverse: 5'-GTGCTGAAGACCTTAGGGCA-3'
Tnfa<br>(TNF-α)	mouse	Forward: 5'- CCCTCACACTCACAAACCAC-3'<br>Reverse: 5'-ACAAGGTACAACCCATCGGC-3'
Adgre1<br>(F4/80)	mouse	Forward: 5'-CTTTGGCTATGGGCTTCCAGTC-3'<br>Reverse: 5'-GCAAGGAGGACAGAGTTTATCGTG-3'
Ifng<br>(IFN-γ)	mouse	Forward: 5'-CGGCACAGTCATTGAAAGCC-3'<br>Reverse: 5'-TGTCACCATCCTTTTGCCAGT-3'
Il6<br>(IL-6)	mouse	Forward: 5'-TGATGGATGCTACCAAACTGGA-3'<br>Reverse: 5'-TGTGACTCCAGCTTATCTCTTGG-3'
Il1b<br>(IL-1β)	mouse	Forward: 5'-CTGGTGTGTGACGTTCCCATTA-3'<br>Reverse: 5'-CCGACAGCACGAGGCTTT-3'
Mip1b<br>(MIP-1β)	mouse	Forward: 5'-AGCTGTGGTATTCCTGACCAAA-3'<br>Reverse: 5'- CTCTCCTGAAGTGGCTCCTC-3'
Ucp1	mouse	Forward: 5'-ACTGCCACACCTCCAGTCATT-3'<br>Reverse: 5'-CTTTGCCTCACTCAGGATTGG-3'
Prdm16	mouse	Forward: 5'-CAGCACGGTGAAGCCATTC-3'<br>Reverse: 5'-GCCGTGTTAAGGAATCTG CTG-3'
Pgc1a<br>(PGC-1α)	mouse	Forward: 5'-AGCCGTGACCACTGACAAC GAG-3'<br>Reverse: 5'-GCTGCATGGTTCTGAGTGCTAAG-3'
Cidea	mouse	Forward: 5'-TGCTCTTCTGTATCGCCCAGT-3'<br>Reverse: 5'-GCCGTGTTAAGGAATCTG CTG-3'
Tbx1	mouse	Forward: 5'-GGCAGGCAGACGAATGTTC-3'<br>Reverse: 5'-GCCGTGTTAAGGAATCTG CTG-3'
Ppara<br>(PPAR-α)	mouse	Forward: 5'-GAGTGCAGCCTCAGCCAA-3'<br>Reverse: 5'- CAGTGGGGAGAGAGGACAGA-3'
Tmem26	mouse	Forward: 5'-ACCCTGTCATCCCACAGAG-3'<br>Reverse: 5'-TGTTTGGTGGAGTCCTAAFFTC-3'
Espt1	mouse	Forward: 5'-ACCCTGATAGCACCAAACGA-3'<br>Reverse: 5'-AGGTCTGCCAGTTCTTGCTC-3'
Cox-4	mouse	Forward: 5'- CTGCCCGGAGTCTGGTAATG-3'<br>Reverse: 5'- CAGTCAACGTAGGGGGTCATC-3'

Cpt-1α	mouse	Forward: 5'- TTGCCCTACAGCTCTGGCATTTCC-3'<br>Reverse: 5'- GCACCCAGATGATTGGGATACTGT-3'
Acadm (MCAD)	mouse	Forward: 5'- ATGACGGAGCAGCCAATGAT-3'<br>Reverse: 5'- TCGTCACCCTTCTTCTCTGCTT-3'Reverse: 5'-3'
Cyto-c	mouse	Forward: 5'- AAATCTCCACGGTCTGTTCGG-3'<br>Reverse: 5'- GGGTATCCTCTCCCCAGGTG-3'
Gnao ($G_{\alpha o}$)	mouse	Forward: 5'-TGGTCTACAGCAACACCATCCAG-3'<br>Reverse: 5'-TTCCATACGACTCACCACGTCA-3'
Gnai1 ($G_{\alpha i1}$)	mouse	Forward: 5'-CTTCTGTGTGGCCCTGAGTGA-3'<br>Reverse: 5'-GATGGACGTGTCTGTAAACCACTTG-3'
Gnai2 ($G_{\alpha i2}$)	mouse	Forward: 5'-ACAACAAGTGGTTCACAGACACCTC-3'<br>Reverse: 5'-TAGCTGGCTGCCTCGTCGTA-3'
Gnai3 ($G_{\alpha i3}$)	mouse	Forward: 5'-TGAAGACTACAGGCATTGTGGAGAC-3'<br>Reverse: 5'-GTTCGGATCTTTGGCCACCTA-3'
Gnas ($G_{\alpha s}$)	mouse	Forward: 5'-CCTCGGCAACAGTAAGAC-3'<br>Reverse: 5'-AGACCTGCTTGTCCTTCTG-3'
Gnaq ($G_{\alpha q}$)	mouse	Forward: 5'-TGGACCGTGTAGCCGACCCT-3'<br>Reverse: 5'-GGCCCCCTACATCGACCATTCTGA-3'
Gna11 ($G_{\alpha 11}$)	mouse	Forward: 5'-CTACTTGGCACTGGCGAGAG-3'<br>Reverse: 5'-GTCACTGGACAGCGTGTTGA-3'
Gna12 ($G_{\alpha 12}$)	mouse	Forward: 5'-CGGCTGGTCAAGATCCTGC-3'<br>Reverse: 5'-ACAAGAACCCTCGAACCCTTA-3'
Gna13 ($G_{\alpha 13}$)	mouse	Forward: 5'-CGGAAACGCTGGTTTGAATGC-3'<br>Reverse: 5'-AGATTCTGTAAGGCGATTGGTCT-3'
Gnβ1 ($G_{\beta}1$)	mouse	Forward: 5'-CTCCTGACACCAGACTGTTTG-3'<br>Reverse: 5'-CATCCCTTCTCGGACATCCC-3'
Gnβ2 ($G_{\beta}2$)	mouse	Forward: 5'-TACACCACTAACAAGGTCCACG-3'<br>Reverse: 5'-CAGATGTTGTCCAAACCCCCA-3'
Gnβ3 ($G_{\beta}3$)	mouse	Forward: 5'-AAGAAGCAGATTGCTGATGCC-3'<br>Reverse: 5'-GTCCCCTTAATGTCCTCCGTG-3'
Gnβ4 ($G_{\beta}4$)	mouse	Forward: 5'-CAGGAGGCTGAACAGCTTCG-3'<br>Reverse: 5'-GGCCCACGGAGTCCATATTA-3'
Gnβ5 ($G_{\beta}5$)	mouse	Forward: 5'-ATGTGCGATCAGACCTTCCTG-3'<br>Reverse: 5'-GGAGCAGTAGTTGAGTTGTTGAG-3'
Gnγ2 ($G_{\gamma}2$)	mouse	Forward: 5'-ACCGCCAGCATAGCACAAG-3'<br>Reverse: 5'-AGTAGGCCATCAAGTCAGCAG-3'
Gnγ5 ($G_{\gamma}5$)	mouse	Forward: 5'-CTCAACCGCGTGAAGGTTTC-3'<br>Reverse: 5'-GGTCTGAAGGGATTCGTACTTG-3'
Gnγ7 ($G_{\gamma}7$)	mouse	Forward: 5'-TCAGGTACTAACAACGTCGCC-3'<br>Reverse: 5'-CAGTAGCCCATCAGGTCTGAC-3'
Gnγ11 ($G_{\gamma}11$)	mouse	Forward: 5'-CCTGCCCTTCACATCGAGG-3'<br>Reverse: 5'-TTGTCTCTGCAACTTGACTTCTT-3'
Gnγ12 ($G_{\gamma}12$)	mouse	Forward: 5'-ATGTCCAGCAAGACGGCAAG-3'<br>Reverse: 5'-GAGGTCGGTATGCCCATCAG-3'
Cxcr4	mouse	Forward: 5'-GAAGTGGGGTCTGGAGACTAT-3'<br>Reverse: 5'-TTGCCGACTATGCCAGTCAAG-3'
Gpr81	mouse	Forward: 5'-TCGTGCTGTCTCATCGAGG-3'<br>Reverse: 5'-TCTTCATGTGAAAGCAGAAGCC-3'
Niacr1	mouse	Forward: 5'-CTGGAGGTTCGGAGGCATC-3'<br>Reverse: 5'-TCGCCATTTTTGGTCATCATGT-3'
Ptger3	mouse	Forward: 5'-CCGGAGCACTCTGCTGAAG-3'<br>Reverse: 5'-CCCCACTAAGTCGGTGAGC-3'
S1pr1	mouse	Forward: 5'-ATGGTGTCCACTAGCATCCC-3'<br>Reverse: 5'-CGATGTTCAACTTGCCTGTGTAG-3'

Sucnr1	mouse	Forward: 5'-TCTTGTGAGAATTGGTTGGCAA-3'<br>Reverse: 5'-CGATGTTCAACTTGCCTGTGTAG-3'
Adora2a	mouse	Forward: 5'-GCCATCCCATTCGCCATCA-3'<br>Reverse: 5'-GCAATAGCCAAGAGGCTGAAGA-3'
Adrb3	mouse	Forward: 5'-GGCCCTCTCTAGTTCCCAG-3'<br>Reverse: 5'-TAGCCATCAAACCTGTTGAGC-3'
Mc2r	mouse	Forward: 5'-ACACCGCAAGAAATAACTCCG-3'<br>Reverse: 5'-AGGAGGACAATCAAGTTCTCCA-3'
Tshr	mouse	Forward: 5'-AGAACTGATCGCAAAAGACACC-3'<br>Reverse: 5'-CCGGATACTGCTCTCATTACAC-3'

1 Krishnan, N. *et al.* PTP1B inhibition suggests a therapeutic strategy for Rett syndrome. *J Clin Invest* **125**, 3163-3177, doi:10.1172/jci80323 (2015).

2 Eguchi, J. *et al.* Transcriptional control of adipose lipid handling by IRF4. *Cell Metab* **13**, 249-259, doi:10.1016/j.cmet.2011.02.005 (2011).

3 Regard, J. B. *et al.* Probing cell type-specific functions of Gi in vivo identifies GPCR regulators of insulin secretion. *J Clin Invest* **117**, 4034-4043, doi:10.1172/jci32994 (2007).

4 Zhu, H. *et al.* Cre-dependent DREADD (Designer Receptors Exclusively Activated by Designer Drugs) mice. *Genesis (New York, N.Y. : 2000)* **54**, 439-446, doi:10.1002/dvg.22949 (2016).
