# Supplementary material for: Adipocyte Gi signaling is essential for maintaining whole-body glucose homeostasis and insulin sensitivity
Source: Nat Commun. 2020 Jun 12;11:2995. doi: 10.1038/s41467-020-16756-x (PMC7293267; doi:10.1038/s41467-020-16756-x)
Supplement: Supplementary file 2 — Reporting Summary [file 41467_2020_16756_MOESM2_ESM.pdf]

nature research

Corresponding author(s): Jurgen Wess

Last updated by author(s): 04-05-2020

Reporting Summary

Nature Research wishes to improve the reproducibility of the work that we publish. This form provides structure for consistency and transparency in reporting. For further information on Nature Research policies, see Authors & Referees and the Editorial Policy Checklist.

Statistics

For all statistical analyses, confirm that the following items are present in the figure legend, table legend, main text, or Methods section.

n/a	Confirmed	
☐	☒	The exact sample size (n) for each experimental group/condition, given as a discrete number and unit of measurement
☐	☒	A statement on whether measurements were taken from distinct samples or whether the same sample was measured repeatedly
☐	☒	The statistical test(s) used AND whether they are one- or two-sided<br>*Only common tests should be described solely by name; describe more complex techniques in the Methods section.*
☐	☒	A description of all covariates tested
☐	☒	A description of any assumptions or corrections, such as tests of normality and adjustment for multiple comparisons
☐	☒	A full description of the statistical parameters including central tendency (e.g. means) or other basic estimates (e.g. regression coefficient) AND variation (e.g. standard deviation) or associated estimates of uncertainty (e.g. confidence intervals)
☐	☒	For null hypothesis testing, the test statistic (e.g. F, t, r) with confidence intervals, effect sizes, degrees of freedom and P value noted<br>*Give P values as exact values whenever suitable.*
☒	☐	For Bayesian analysis, information on the choice of priors and Markov chain Monte Carlo settings
☒	☐	For hierarchical and complex designs, identification of the appropriate level for tests and full reporting of outcomes
☒	☐	Estimates of effect sizes (e.g. Cohen's d, Pearson's r), indicating how they were calculated

Our web collection on statistics for biologists contains articles on many of the points above.

Software and code

Policy information about availability of computer code

Data collection	Prism, Excel
Data analysis	Prism, Excel

For manuscripts utilizing custom algorithms or software that are central to the research but not yet described in published literature, software must be made available to editors/reviewers. We strongly encourage code deposition in a community repository (e.g. GitHub). See the Nature Research guidelines for submitting code & software for further information.

Data

Policy information about availability of data

All manuscripts must include a data availability statement. This statement should provide the following information, where applicable:

- Accession codes, unique identifiers, or web links for publicly available datasets
- A list of figures that have associated raw data
- A description of any restrictions on data availability

Source data for all figures are provided with the paper. All other data supporting the findings of this study are available from the authors upon request.

Field-specific reporting

Please select the one below that is the best fit for your research. If you are not sure, read the appropriate sections before making your selection.

☒ Life sciences ☐ Behavioural & social sciences ☐ Ecological, evolutionary & environmental sciences

For a reference copy of the document with all sections, see nature.com/documents/nr-reporting-summary-flat.pdf

Life sciences study design

All studies must disclose on these points even when the disclosure is negative.

Sample size	Sample size was chosen based on prior experience of the investigators with similar experiments previously published. The authors have published numerous peer-reviewed papers demonstrating clear positive findings with similar sample sizes for the types of experiments included.
Data exclusions	No data points were excluded from the analysis of any of the experimetns.
Replication	All experimental findings were reproduced in several independent experiments.
Randomization	Randomization was performed by blinding investigators to genotype and allowing them to choose each subject blindly.
Blinding	Randomization was performed by blinding investigators to genotype and allowing them to choose each subject blindly. For studies using pharmacological agents, the investigator was aware of the agent being used, but was not aware of the genotypes of the animals used. Investigators were not aware of the specific group to which an animal was assigned to when doing the experiment or until after completion of the experiment.

Reporting for specific materials, systems and methods

We require information from authors about some types of materials, experimental systems and methods used in many studies. Here, indicate whether each material, system or method listed is relevant to your study. If you are not sure if a list item applies to your research, read the appropriate section before selecting a response.

Materials & experimental systems

n/a	Involved in the study	
☐	☒	Antibodies
☒	☐	Eukaryotic cell lines
☒	☐	Palaeontology
☐	☒	Animals and other organisms
☒	☐	Human research participants
☒	☐	Clinical data

Methods

n/a	Involved in the study	
☒	☐	ChIP-seq
☒	☐	Flow cytometry
☒	☐	MRI-based neuroimaging

Antibodies

Antibodies used

Reagent - Source - Catalog # (identifier)
Antibodies
Phospho-Insulin Receptor beta (Tyr1150/1151) Cell Signaling 3024
Phospho-Insulin Receptor beta (Tyr1146) Cell Signaling 3021
Insulin Receptor beta Cell Signaling 3020
Phospho-IRS1 (Ser612) Cell Signaling 3203
IRS1 Cell Signaling 2382
PI3 Kinase p85 Cell Signaling 4257
Phospho-PI3 Kinase p85 (Tyr458)/p55 (Tyr199) Cell Signaling 4228
Phospho-Akt (Thr308) Cell Signaling 2965
Phospho-Akt (Ser473) Cell Signaling 4060
Akt Cell Signaling 9272
Phospho-FoxO1 (Ser256) Cell Signaling 9461
FoxO1 Cell Signaling 2880 (clone C29H4)
Phospho-GSK-3beta(Ser9) Cell Signaling 9336
GSK-3alpha/beta (D75D3) Cell Signaling 5676
Phospho-HSL (Ser563) Cell Signaling 4139
Phospho-HSL (Ser660) Cell Signaling 4126
HSL Cell Signaling 4107
Phospho-ATGL (Ser406) Abcam ab135093
ATGL Abcam 2138
Phospho-AS160 (Thr642) Millipore Sigma ABS271
AS160 Millipore Sigma 07-741
Phospho-NF-κB p65 Cell Signaling 3033
NF-κB p65 Cell Signaling 8242
S1-PTX EpiGentek A53718
Beta-tubulin Cell Signaling 86298
F4/80 Abcam Ab6640

	UCP-1 Abcam Ab10983<br>Beta-actin Cell Signaling 4970
Validation	Antibodies were validated by the manufacturers.

Animals and other organisms

Policy information about studies involving animals; ARRIVE guidelines recommended for reporting animal research

Laboratory animals	Male mice (C57BL6 background) older than 8 weeks
Wild animals	N/A
Field-collected samples	N/A
Ethics oversight	All animal studies were approved by the NIDDK institutional Animal Care and Use Committee.

Note that full information on the approval of the study protocol must also be provided in the manuscript.
